# Mechanisms of traditional Chinese medicine interventions in the gastric ‘inflammation-to-carcinoma’ cascade: advances in *in vitro* models from monolayer cells to organoids

**DOI:** 10.3389/fphar.2026.1906847

**Published:** 2026-10-13

**Authors:** Xiaoling Zhang, Tiangang Wang, Xusen Zeng, Xin Zhou, Long Zhao

**Affiliations:** Department of Spleen and Stomach Diseases, The Affiliated Traditional Chinese Medicine Hospital of Southwest Medical University, Luzhou, Sichuan, China

**Keywords:** Correa cascade, gastric organoids, GES-1 cells, *in vitro* models, inflammation to carcinoma transition, traditional Chinese medicine

## Abstract

Gastric cancer remains a leading cause of cancer-related mortality worldwide. The Correa cascade, progressing from chronic gastritis through precancerous lesions to adenocarcinoma, provides the framework for understanding gastric mucosal stepwise malignant transformation. Traditional Chinese Medicine (TCM) formulas, such as Zuojin Pills and Wuji Pills, have been historically used to treat gastric disorders including epigastric pain and acid regurgitation, yet their mechanisms of action in blocking the inflammation-to-carcinoma transition remain incompletely understood. The mechanistic diversity of TCM presents challenges for *in vitro* modeling, and existing reviews have not systematically addressed how model selection should align with specific research questions across different stages of the cascade. This review examines the evolving landscape of *in vitro* models used in TCM research on gastric mucosal inflammation-to-carcinoma transition, highlighting the paradigm shift from two-dimensional (2D) monolayer GES-1 cell cultures to three-dimensional gastric organoids. We analyzed recent studies conducted across multiple model platforms using various acute or chronic pathogenic factors, with all botanical drugs validated taxonomically through authoritative sources. The GES-1 cell model offers low cost, high throughput, and genetic homogeneity, making it suitable for initial screening of active metabolites from botanical drugs and for dissecting molecular pathways, particularly those involving Nrf2, NF-κB, PI3K/AKT, and MAPK signaling in antioxidative stress, anti-inflammatory, and anti-apoptotic mechanisms. In contrast, gastric organoids preserve three-dimensional architecture, cellular diversity, and patient-specific genetic backgrounds, enabling functional assessment of barrier integrity, stem cell-driven regeneration, and malignant transformation at the tissue level. To address the need for evidence differentiation, we introduce a hierarchical framework (Model Evidence Level) in which GES-1 cells serve for entry-level target discovery and patient-derived organoids for higher-level tissue validation. Based on this framework, we provide stage-specific model selection recommendations corresponding to the distinct phases of the Correa cascade: acute inflammation, chronic atrophy, intestinal metaplasia, dysplasia, and gastric cancer. This review offers a practical, evidence-stratified pathway for *in vitro* model selection in TCM intervention studies, bridging traditional ethnopharmacological knowledge with modern pharmacological validation to strengthen the translational relevance of preclinical findings.

## Graphical Abstract

**Figure:**
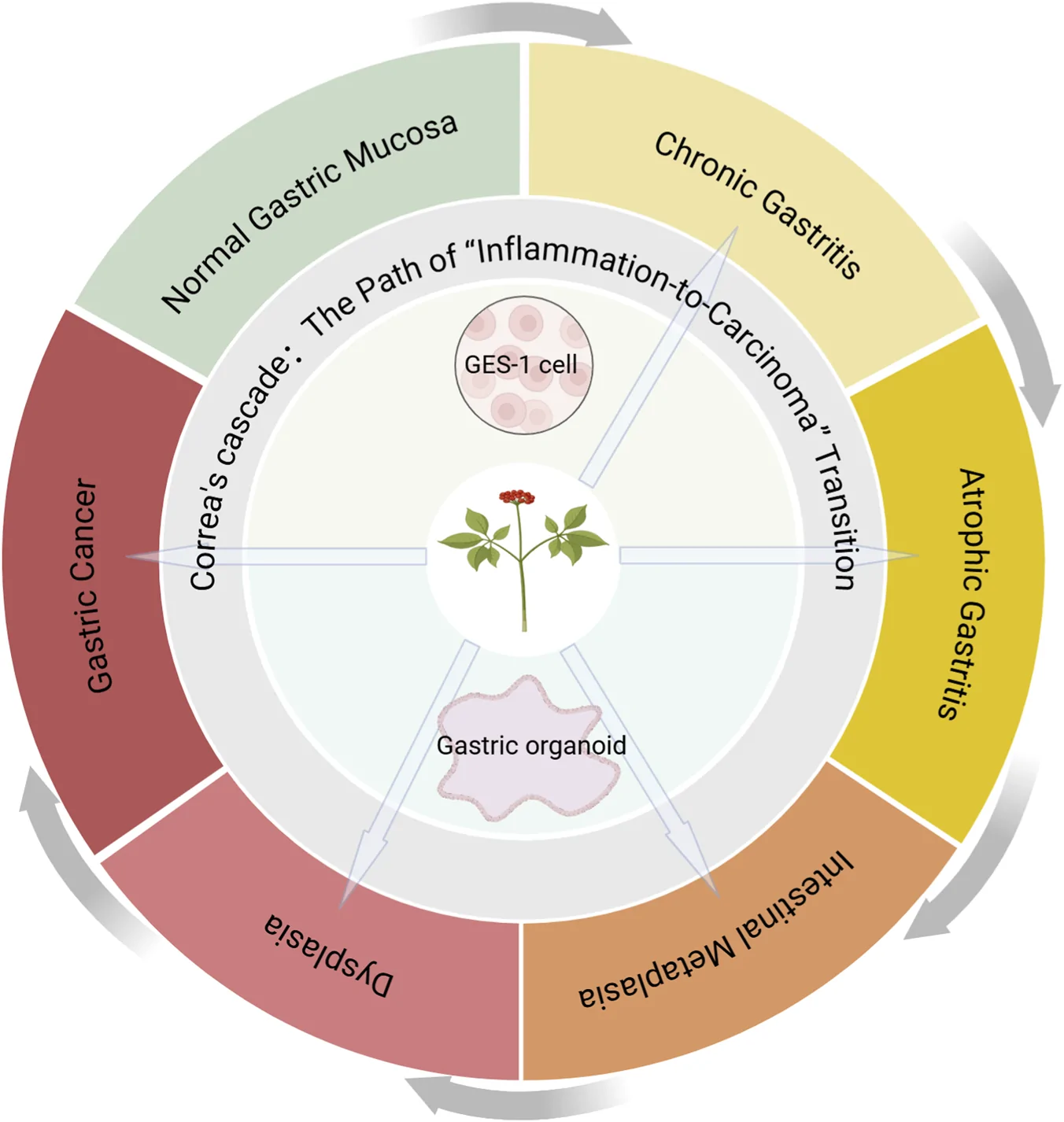
Created in BioRender. Zhang et al. (2026) https://BioRender.com/p3stked.

## 1 Introduction

Gastric cancer is the fifth most common cancer and the fifth leading cause of cancer-related death worldwide (Duan Y. et al., 2025). The normal gastric mucosa progressively transitions from inactive or chronic active gastritis to precancerous lesions and ultimately to gastric adenocarcinoma. This continuous pathological process is known as the Correa cascade (Liao W. et al., 2025). As the initiating stage of this cascade, inflammatory diseases of the gastric mucosa pose a serious threat to human health. A systematic analysis of the global burden of disease indicated that in 2021, there were 27.2 million patients with gastritis and duodenitis worldwide (Wang L. et al., 2025). Therefore, early intervention in inflammation-related gastric mucosal diseases is of great significance for delaying the inflammation-to-carcinoma transition process and reducing the incidence of gastric cancer.

The integrity of the gastric mucosal epithelium serves as the structural foundation for defending against damaging factors and maintaining gastric homeostasis. The gastric mucosal epithelium comprises multiple functionally specialized cell types. Surface mucous cells secrete mucus rich in bicarbonate, forming a physicochemical barrier. Chief cells secrete pepsinogen, which participates in protein digestion. Parietal cells secrete hydrochloric acid and intrinsic factor, maintaining the acidic environment and facilitating vitamin B12 absorption. Endocrine cells and G cells coordinate gastric gland function through secretion of hormones including histamine, somatostatin, and gastrin (Engevik et al., 2020). Stem cells located at the gastric gland isthmus possess proliferative and differentiation potential. They continuously renew damaged epithelial cells and maintain mucosal homeostasis. When damaging factors—including *Helicobacter pylori* infection, non-steroidal anti-inflammatory drugs (NSAIDs), alcohol, or stress—persist, mucus secretion decreases or gastric acid secretion becomes imbalanced. Damaging agents then penetrate the mucus barrier and directly attack epithelial cells. Cellular damage triggers inflammatory responses. If damage is mild and repair occurs in a timely manner, the mucosa can recover its integrity. If damage persists or repair is insufficient, inflammation progresses from superficial gastritis to erosive gastritis, atrophic gastritis, dysplasia, and ultimately gastric cancer. Thus, the dynamic balance between damage and repair of gastric mucosal epithelial cells is a critical determinant of the trajectory of inflammation-to-carcinoma transition. Therefore, in-depth analysis of the response mechanisms of gastric mucosal epithelial cells to damaging factors, the molecular events of cellular damage and repair, and the protective effects of pharmacological interventions is essential for elucidating the pathogenesis of gastric mucosal diseases and developing effective intervention strategies.

Current clinical strategies for treating gastric mucosal diseases include *H. pylori* eradication, gastric acid suppression, and mucosal protection. Antibiotics, proton pump inhibitors, and gastric mucosal protectants are widely used. In recent years, TCM has attracted increasing research attention due to its multi-component nature. TCM may exert pharmacological effects through multiple pathways, demonstrating unique advantages in treating inflammatory gastric mucosal diseases. Zuojin Pills (ZJP; composed of *Coptis chinensis* Franch. [Ranunculaceae] and *Tetradium ruticarpum* (A.Juss.) T.G.Hartley [Rutaceae] in a 6:1 ratio), a classic TCM formula commonly used in digestive system disorders, has shown significant efficacy in alleviating clinical symptoms such as gastric pain and acid reflux. Studies have demonstrated that Zuojin Pills can reduce serum inflammatory cytokine levels in a chronic atrophic gastritis rat model. They also significantly ameliorate gastric inflammatory lesions and promote gastric mucosal epithelial cell proliferation while attenuating *H. pylori*-or drug-induced cellular damage *in vitro* (Tong et al., 2021; Wen et al., 2022). Wuji Pills (WJP; composed of *C. chinensis* Franch. [Ranunculaceae], *T. ruticarpum* (A.Juss.) T.G.Hartley [Rutaceae], and *Paeonia lactiflora* Pall. [Paeoniaceae] in a 6:1:6 ratio), a TCM patent medicine approved for marketing in China, have been shown to alleviate inflammation and oxidative stress injury. They also inhibit apoptotic signaling pathways and exert therapeutic effects on ethanol-induced gastric ulcers (Wu et al., 2024). These findings suggest that TCM may exert unique value in early intervention of the inflammation-to-carcinoma transition by regulating the fate of gastric mucosal epithelial cells.

*In vitro* culture models are indispensable tools for elucidating how gastric epithelial cells respond to damaging factors and for assessing the effects of TCM interventions. In recent years, this field has undergone a paradigm shift from “monolayer cell culture” to “three-dimensional organoid reconstruction.” The human gastric epithelial cell line GES-1, due to its ease of use and homogeneous genetic background, has become the mainstream platform for initial screening of molecular mechanisms. For example, in studies using GES-1 cells as an *in vitro* model, key active metabolites from the rhizomes of *C. chinensis* Franch. (Ranunculaceae) and the stem barks of *Magnolia officinalis* Rehder and E.H.Wilson (Magnoliaceae) were found to enhance cell viability and inhibit apoptosis, thereby alleviating inflammatory responses in rats with chronic gastritis (Song et al., 2023). Likewise, gentiopicroside from *Gentiana macrophylla* Pall. (Gentianaceae) improves GES-1 cell survival by regulating matrix metalloproteinase-10 (MMP-10) and pERK1/2 signaling, alleviating ethanol-induced gastritis in mice (Chang et al., 2021). However, as GES-1 cells are cultured in monolayers, it is difficult to replicate the three-dimensional structure and intercellular interactions of the *in vivo* gastric mucosa. Through three-dimensional cell culture techniques and *in vitro* stem cell expansion, gastric organoids can mimic the three-dimensional structure and cellular composition of the native gastric mucosa. Their biological characteristics closely resemble those of the original tissue, and they retain the ability to self-renew and proliferate *in vitro*, providing a more clinically relevant model for validating tissue-level functions and conducting personalized pharmacodynamic evaluations (Huo et al., 2023).

Both model types have been applied in TCM research in recent years. Existing reviews have largely focused on organoid culture techniques, pharmacodynamic studies of TCM at specific gastric mucosal lesion stages, or mechanistic pathways of TCM intervention in the Correa cascade. However, few have compared GES-1 monolayer cells and gastric organoids from the perspective of model selection and integration with respect to applicable stages, levels of evidence, and complementary relationships. This review distinguishes the application scenarios of acute versus chronic models in GES-1 cells. We clarify that chronically induced precancerous models—established using MNNG, deoxycholic acid, and similar agents—serve as preliminary screening tools rather than tissue-level validation platforms. On this basis, we propose a hierarchical research paradigm. In this paradigm, GES-1 cells are used to identify molecular targets, and gastric organoids are used to verify tissue-level functions. Taking this research logic as the central theme, we summarize the model selection strategies applicable to every stage of the Correa cascade. We emphasize the logic of which research questions different models can address. This review aims to provide a more operational theoretical basis for methodological selection of *in vitro* approaches in TCM intervention against gastric mucosal inflammation-to-carcinoma transition.

## 2 Literature search strategy

This article is a narrative review rather than a systematic review. Therefore, it does not follow the strict literature screening process prescribed by the PRISMA guidelines. However, to ensure transparency and traceability of the retrieval process and to minimize selection bias, we conducted a structured literature search in PubMed, Web of Science, and SinoMed databases. The search covered the period from 1 January 2021, to 30 June 2026.

The search keywords covered the following three dimensions, combined with AND between dimensions and OR within each dimension: ① TCM intervention: traditional Chinese medicine, traditional medicine, Chinese herbal medicine, Chinese botanical drugs, Chinese herbal formulas, Chinese patent medicines; ② Disease/pathological stages: gastric diseases, gastric mucosal injury, gastritis, atrophic gastritis, gastric cancer, gastric neoplasm, intestinal metaplasia, dysplasia; ③ Research models: GES-1 cells, monolayer cells, gastric organoids, patient-derived organoids, gastric precancerous organoids, gastric cancer organoids, *in vitro* studies, *in vitro* models. The same logical structure was applied across the three databases, with expressions adjusted according to each database’s syntax rules. The search was limited to English-language publications.

Inclusion criteria: Study type: original experimental studies involving molecular mechanisms. Model: studies using the GES-1 cell line (including normal culture, induced injury, genetic intervention, etc.) or gastric organoid models (including patient-derived, *H. pylori*-infected, precancerous, and gastric cancer organoids), or comparative studies using both model types. Intervention: studies using active TCM metabolites, TCM extracts, TCM active fractions, TCM formulas, TCM patent medicines, or TCM-containing serum as interventions. Chemically synthesized drugs, biologics, and natural products not derived from TCM sources were not considered core inclusion targets. Topic: studies related to gastric mucosal inflammation-to-carcinoma transition (Correa cascade), including but not limited to gastric mucosal protection, anti-inflammation, anti-oxidative stress, anti-apoptosis, anti-*H. pylori*, reversal of precancerous lesions, blockade of malignant transformation, and inhibition of gastric cancer cell proliferation.

Exclusion criteria: Conference abstracts, case reports, reviews, systematic reviews, meta-analyses, and other non-original studies; studies using only animal models or clinical tissue samples without *in vitro* models; studies using non-gastric cell lines or using gastric epithelial cell lines other than GES-1 as the sole model; interventions involving chemical drugs, biologics, natural products not derived from TCM sources, or synthetic compounds not derived from natural products; studies not addressing mechanisms of gastric mucosal inflammation-to-carcinoma transition, focusing only on pharmacokinetics, chemical structures, or formulation modification without pharmacodynamic data, or relying solely on network pharmacology predictions without experimental validation; studies with major experimental design flaws, incomplete result reporting, non-peer-reviewed publications, duplicate publications, or studies for which the full text could not be obtained.

The initial screening yielded 242 articles, of which 49 were ultimately included as highly relevant to the topic of this review.

## 3 Key inducers of the inflammation-to-carcinoma transition in the gastric mucosa

To simulate the complex process of gastric mucosal inflammation-to-carcinoma transition *in vitro*, specific damaging factors are required to stimulate cells or organoids to model different stages of the disease. Depending on the research objectives, these factors can be divided into two categories. The first comprises stimuli that mimic acute injury, inducing oxidative stress, inflammatory responses, or programmed cell death. The second comprises inducers of chronic accumulation and carcinogenic progression. These factors drive cells from inflammation toward metaplasia and dysplasia through sustained low-dose exposure. They serve as key tools for validating the effects of TCM in blocking inflammation-to-carcinoma transition (Figure 1).

**FIGURE 1:**
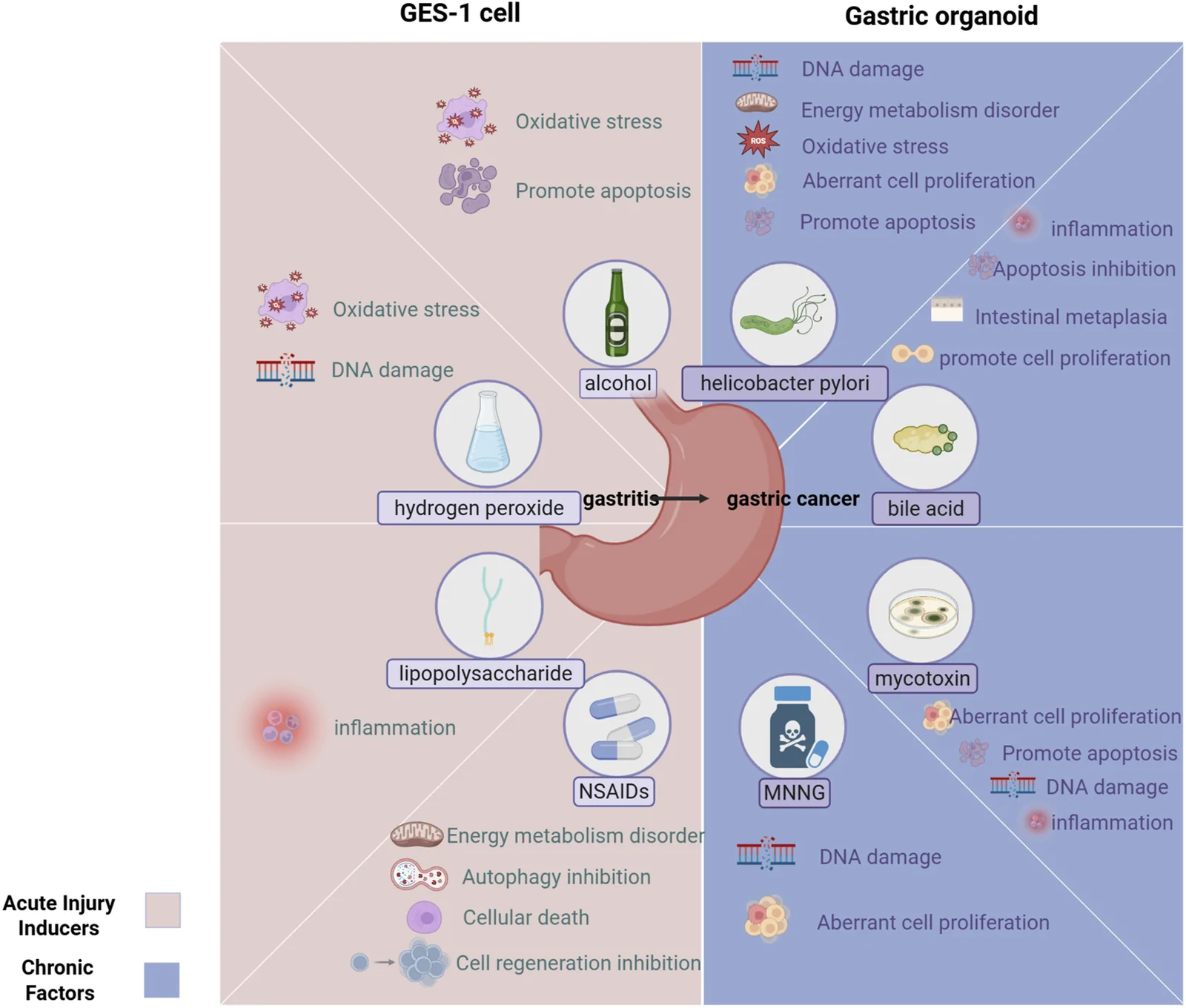
Acute and chronic injury factors are employed in *in vitro* models to mimic the distinct stages of gastric mucosal inflammation-to-carcinoma transition. Created in BioRender. Zhang et al. (2026) https://BioRender.com/p3stked.

### 3.1 Acute injury factors

#### 3.1.1 Ethanol

Ethanol rapidly penetrates the gastric mucosal barrier and directly damages gastric mucosal epithelial cells. Through oxidation by cytochrome P450 2E1, ethanol generates acetaldehyde and reactive oxygen species (ROS), triggering oxidative stress and lipid peroxidation (Contreras-Zentella et al., 2022). Ethanol also induces rapid death of GES-1 cells in a concentration-dependent manner and activates phosphorylation of key mediators in the necroptosis pathway (Liu et al., 2021). Ethanol treatment is a common method for establishing an acute injury model in GES-1 cells, mimicking acute gastritis and gastric ulcers. It is used to evaluate the immediate protective effects of TCM against oxidative stress and apoptosis.

#### 3.1.2 Hydrogen peroxide (H_2_O_2_)

H_2_O_2_ is a metabolic byproduct that induces the generation of oxygen free radicals and ROS release. This triggers oxidative stress and damages biological macromolecules. Studies have found that H_2_O_2_ stimulation induces lipid peroxidation in GES-1 cells, generating endogenous aldehydes primarily consisting of 4-hydroxynonenal (4-HNE). 4-HNE interacts with nucleic acids to induce DNA damage. It also affects protein modification and cell signaling, ultimately disrupting cellular metabolism (Duan et al., 2016). This mechanism provides a theoretical basis for *in vitro* screening of TCM active metabolites with aldehyde detoxification and anti-oxidative stress effects.

#### 3.1.3 Lipopolysaccharide (LPS)

Microbial infection and tissue damage are two major drivers of gastric mucosal inflammation. As the primary component of Gram-negative bacterial cell walls, LPS mimics pathogen invasion. It activates the nuclear factor-κB (NF-κB) signaling pathway in GES-1 cells through toll-like receptor 4 (TLR4), leading to acute inflammatory injury (Yang et al., 2020). Therefore, LPS can be used to study TCM intervention at the initiation of inflammatory signaling. Additionally, LPS treatment is often combined with co-culture systems of GES-1 cells and immune cells. This simulates epithelial–immune interactions and provides an entry point for exploring microenvironmental factors in inflammation-to-carcinoma transition.

#### 3.1.4 Nonsteroidal anti-inflammatory drugs (NSAIDs)

NSAIDs, represented by aspirin, inhibit cyclooxygenase and rapidly deacetylate to salicylic acid. They reduce cytoplasmic adenosine triphosphate (ATP), stimulate sodium transport, and increase proton dissipation in surface epithelial cells. These effects lead to cellular energy metabolic disorders (Kauffman, 1989). In this state, large amounts of ROS are generated, triggering lipid peroxidation and accumulation of oxygen free radicals. These directly damage lipids, DNA, and proteins, ultimately leading to cell death (Pal et al., 2010). Hernández et al. (Hernández et al., 2016) found that aspirin also eliminates the self-protective mechanisms of gastric mucosal epithelial cells by inhibiting autophagy. This exacerbates the transition from oxidative damage to irreversible cell death. Inhibition of gastric mucosal epithelial cell proliferation through suppression of epidermal growth factor is another mechanism by which NSAIDs induce gastritis. This prevents mucosal repair through cell regeneration (Pai et al., 2001). NSAIDs are commonly used to establish *in vitro* models of acute drug-induced gastric injury, enabling evaluation of TCM regulation of autophagy.

### 3.2 Factors contributing to chronic accumulation and carcinogenesis

#### 3.2.1 Helicobacter pylori

*Helicobacter pylori* is a key factor in the inflammation-to-carcinoma transition of the gastric mucosa. After entering the digestive tract, *H. pylori* first penetrates the gastric mucus layer through flagellar motility and the ammonia-producing action of urease, reaching and attaching to gastric mucosal epithelial cells via adhesion factors. The ammonia produced during this process can also induce cytotoxicity (Toh and Wilson, 2020). Its various virulence factors, such as cytotoxin-associated protein A (CagA), vacuolating cytotoxin A (VacA), and neutrophil-activating protein (NAP), play key roles in cytotoxicity. Among these, VacA can disrupt mitochondria and impair energy production (Seeger et al., 2023); it can also induce vacuolization, promote apoptosis, and inhibit GES-1 cell proliferation (Wang et al., 2022). Studies have shown that *H. pylori* induces excessive DNA damage in human gastric mucosal epithelial cells. Following DNA damage, the DNA damage response (DDR) mechanism must be activated to maintain the integrity of the human cellular genome; however, CagA can lead to reduced levels of key DDR components, impairing DDR function, which may further contribute to the development of gastric cancer (Kontizas et al., 2020). NAP primarily causes damage to human gastric epithelial cells by inducing oxidative stress through the production of ROS by neutrophils and monocytes (Fu and Lai, 2022). The co-culture model of *H. pylori* and gastric epithelial cells is a classic system for studying the inflammation-to-carcinoma transition. In particular, the long-term infection model using CagA-positive *H. pylori* strains can simulate the critical transition from chronic gastritis to gastric mucosal atrophy and intestinal metaplasia, serving as a core tool for validating the anti-*H. pylori* effects of TCM and its ability to block malignant transformation.

#### 3.2.2 Chemical carcinogens

Nitrites are classified as Group 2A carcinogens. When excessive nitrite is ingested, it is converted to nitrosamine compounds under acidic gastric conditions, particularly in the presence of vitamin C deficiency. N-methyl-N′-nitro-N-nitrosoguanidine (MNNG) induces DNA damage profiles in gastric mucosal epithelial cells similar to those caused by dimethylnitrosamine (Arabski et al., 2006). It triggers genomic instability and drives the progression of gastric mucosal epithelial cells from simple damage toward malignant transformation. This process is highly consistent with environmental triggers of human gastric carcinogenesis. Liu et al. (2024) found in both *in vivo* and *in vitro* experiments that long-term low-concentration MNNG exposure upregulated methyltransferase-like protein 3 (*METTL3*) expression. This mediated epithelial–mesenchymal transition (EMT) and promoted gastric carcinogenesis. Models of gastric precancerous lesions and gastric cancer cells induced by MNNG are used to study the effects of TCM on DNA damage repair and cell cycle regulation. In gastric organoids, long-term MNNG treatment can induce tissue-level changes such as glandular structural disorganization and abnormal proliferation. This enables validation of whether TCM truly possesses the potential to reverse precancerous lesions.

#### 3.2.3 Bile acids

Bile reflux is a major contributor to gastric mucosal intestinal metaplasia and is closely associated with the development of precancerous lesions and gastric cancer. Deoxycholic acid (DCA) is the primary bile acid component in bile reflux. In gastric epithelial cells, DCA promotes proliferation and inhibits apoptosis, upregulates pro-inflammatory cytokines and intestinal metaplasia markers, and promotes STAT3 phosphorylation (Jin et al., 2022), indicating a close association between bile reflux and gastrointestinal metaplasia. Wang S. et al. (2022) found that treatment with taurodeoxycholic acid (TDCA) significantly upregulated the levels of the proliferation marker Ki67 and the cell cycle regulator Cyclin D1 in GES-1 cells, suggesting that TDCA may promote cell proliferation by accelerating the cell cycle; simultaneously, TDCA increased cellular IL-6 levels, activated the IL-6/JAK1/STAT3 pathway, and thereby promoted gastric cancer development. These factors are commonly used to establish *in vitro* models of bile reflux gastritis and intestinal metaplasia, offering unique value for studying TCM interventions at the intestinal metaplasia stage of the inflammation-to-carcinoma transition.

#### 3.2.4 Environmental toxins

Various mycotoxins, such as aflatoxins, deoxynivalenol (DON), and ochratoxins, can cause damage to gastric mucosal epithelial cells both *in vivo* and *in vitro*. Aflatoxin G1 (AFG1) is one of the most common mycotoxins; its ingestion can induce TNF-α-mediated gastritis, upregulate cytochrome P450 2E1 (CYP2E1), and promote DNA damage in gastric mucosal epithelial cells (Wu X. et al., 2023). In a study using GES-1 cells as a model, DON was shown to enter the cell nucleus via simple diffusion, inducing DNA damage, apoptosis, and G2 phase arrest (Hou et al., 2023). Furthermore, ochratoxin A (OTA) enhances glycolysis mediated by the mTOR/HIF-1α pathway and activates inflammatory responses in GES-1 cells (Wang Y. et al., 2024). Long-term OTA exposure also induces malignant transformation of GES-1 cells through intracellular ROS production and activation of the Wnt/β-catenin signaling pathway (Jia et al., 2016). Models of long-term, low-dose mycotoxin exposure may be used to simulate the chronic cumulative effects of environmental toxins in the inflammation-to-carcinoma transition.

In summary, injury factors differ in duration and pathological effects, making them suitable for different Correa cascade stages. Acute factors are easy to manipulate and short-acting, ideal for acute injury models and preliminary screening of TCM protective mechanisms in monolayer GES-1 cells. Chronic cumulative factors are better suited to three-dimensional long-term culture, such as gastric organoids, to model precancerous lesions and gastric cancer and to observe TCM effects on differentiation, proliferation, and malignant transformation at the tissue level. Rational selection of injury factors and exposure protocols is therefore prerequisite for reliable *in vitro* modeling of TCM mechanisms.

## 4 TCM intervention mechanisms based on GES-1 cell models

All studies reviewed in this section are based on GES-1 monolayer cells, these represent the entry-level evidence tier (immortalized cell lines) in our hierarchical framework, suitable for molecular target discovery and mechanistic screening, but requiring further validation in higher-order models.

### 4.1 Maintenance of cellular redox balance and antioxidant defense

Acute stimulating factors such as ethanol and H_2_O_2_ induce massive ROS production in gastric mucosal epithelial cells. This triggers oxidative stress and attacks lipids, proteins, and DNA—a critical step in acute gastric mucosal injury. Recent studies have shown that TCM can exert cytoprotective effects through anti-oxidative stress. For example, AVLP-2, a novel acidic polysaccharide from the dried mature fruits of *Amomum villosum* Lour. (Zingiberaceae), reduced intracellular ROS levels, increased superoxide dismutase (SOD) and glutathione (GSH) activities, and decreased malondialdehyde (MDA) content in ethanol-induced GES-1 cells (Liu et al., 2022). This preliminarily validated the antioxidant effects of TCM active metabolites in GES-1 cells. However, a deeper question is whether TCM can enhance endogenous antioxidant capacity by intervening in the cell’s own antioxidant defense system.

At the molecular level, the Keap1/Nrf2 pathway is the core regulatory axis maintaining cellular redox balance. Keap1 is a negative regulator of Nrf2. Under normal conditions, Keap1 binds Nrf2 and mediates its ubiquitination and degradation. Upon oxidative stimulation, Keap1 cysteine residues are modified. This leads to conformational changes in Keap1 and inhibition of the proteasomal degradation mechanism. Newly synthesized Nrf2 then enters the nucleus, binds to the antioxidant response element (ARE), and activates downstream gene transcription, thereby activating the antioxidant defense system (Adinolfi et al., 2023). Heme oxygenase-1 (HO-1) and NADPH quinone oxidoreductase 1 (NQO1) are two key effector molecules downstream of Nrf2 (Acevedo et al., 2024). HO-1 catalyzes the degradation of the pro-oxidant molecule heme into biliverdin/bilirubin and carbon monoxide. These products possess significant antioxidant and anti-inflammatory effects (Durante, 2020). NQO1 maintains the reduced state of coenzyme Q and vitamin E, blocking the chain reaction of lipid peroxidation (Ross and Siegel, 2021). In GES-1 cells, various TCM formulas and their active metabolites exert antioxidant effects through regulation of this pathway. Xiaojianzhong Decoction (XJZ; composed of *Neolitsea cassia* (L.) Kosterm [Lauraceae], *P. lactiflora* Pall. [Paeoniaceae], *Glycyrrhiza uralensis* Fisch. ex DC. [Fabaceae], *Zingiber officinale* Rosc. [Zingiberaceae], *Ziziphus jujuba* Mill. [Rhamnaceae], and *Saccharum granorum*) activates Nrf2 via p62-mediated signaling (Chen et al., 2022). Costunolide from *Dolomiaea costus* (Falc.) Kasana and A.K.Pandey (Asteraceae) directly promotes Nrf2 nuclear translocation (Wang R. et al., 2024). Xiangshao Decoction (XST; composed of *Cyperus rotundus* L. [Cyperaceae], *P. lactiflora* Pall. [Paeoniaceae], *Bletilla striata* (Thunb.) Rchb.f. [Orchidaceae], *Atractylodes macrocephala* Koidz. [Asteraceae], *Sepiella maindroni* de Rochebrune [Sepiidae], *Ligusticum chuanxiong* Hort. [Apiaceae], *Citrus aurantium* L. [Rutaceae], *G. uralensis* Fisch. ex DC. [Fabaceae], and *Gypsum fibrosum*) and short-chain fructo-oligosaccharides (scFOS) from the roots of *Cichorium intybus* L. (Asteraceae) promote Nrf2 expression by inhibiting the upstream negative regulator KEAP1 (Gong et al., 2023; Chen et al., 2024). These agents act at different nodes of the Keap1/Nrf2 pathway. The ultimate effect is upregulation of antioxidant genes including HO-1, NQO1, and SOD, and clearance of ROS and MDA, thereby alleviating oxidative stress injury.

Furthermore, the Nrf2 pathway is subject to bidirectional regulation by MAPK family members (p38, ERK, JNK). Related studies indicate that moderate activation of ERK and p38 can mediate Nrf2 nuclear translocation and HO-1 transcriptional activation. However, excessive activation of JNK and p38 weakens Nrf2 transcriptional activity by promoting its cytoplasmic retention (Yao et al., 2007; Ma Y. et al., 2022). In ethanol-induced GES-1 oxidative stress models, piperine from the fruits of *Piper nigrum* L. (Piperaceae) downregulated the negative regulator Keap1 and activated the Nrf2/HO-1 axis. It also reduced the mRNA transcription levels of JNK and p38. This suggests that its antioxidant effects may involve simultaneous regulation of Nrf2 transcriptional activity and upstream MAPK signals (Duan et al., 2022).

Collectively, these findings indicate that TCM can upregulate endogenous antioxidant genes in gastric mucosal epithelial cells through the Keap1/Nrf2/ARE pathway. This enhances their antioxidant capacity rather than relying on direct neutralization by exogenous antioxidants (Figure 2).

**FIGURE 2:**
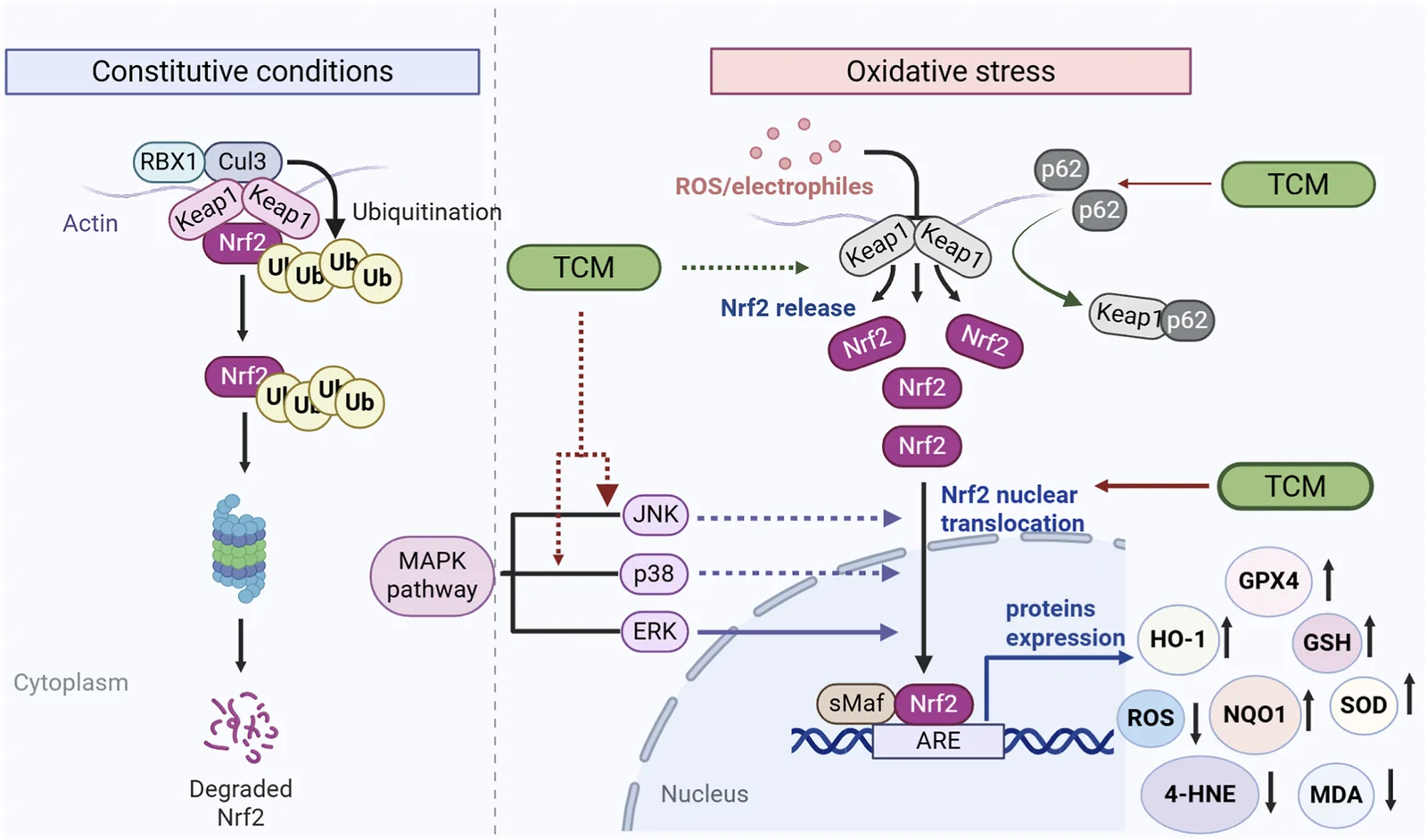
TCM protects gastric mucosal cells against oxidative stress injury via the Keap1/Nrf2 pathway. Created in BioRender. Zhang et al. (2026) https://BioRender.com/p3stked.

### 4.2 Regulation of inflammatory signaling pathways

When ROS production exceeds cellular clearance capacity, unneutralized ROS activate inflammatory pathways, further triggering inflammatory cascades (Xu et al., 2025). The NF-κB signaling pathway, as the core transcriptional hub regulating inflammatory cytokines, is the primary target of TCM anti-inflammatory effects. In the resting state, p50/p65 dimers are retained in the cytoplasm through binding to IκBα. Upon stimulation by ethanol, LPS, or other factors, the IKK complex (with IKKβ at its core) phosphorylates IκBα and mediates its ubiquitination and degradation. This promotes the release of p50/p65 dimers and their entry into the nucleus, initiating transcription of pro-inflammatory cytokines and promoting inflammation (Guo et al., 2024).

TCM can reduce cytokine production by interfering with p65 nuclear translocation. In ethanol-stimulated GES-1 cells, Dangshen Huangjiu from the roots of *Codonopsis pilosula* (Franch.) Nannf. (Campanulaceae) and Chinese Yam aqueous extract (CYW) from the rhizomes of *Dioscorea opposita* Thunb. (Dioscoreaceae) inhibited p65 nuclear translocation by downregulating IKKβ-mediated IκBα phosphorylation. This blocks inflammatory gene transcription at the source (Xu Q. et al., 2023; Xie Y. et al., 2024). Quercetin, from *Citrus reticulata* Blanco (Rutaceae) and other plant sources, downregulated pro-inflammatory cytokine expression by inhibiting TNF-α-induced p65 nuclear translocation in GES-1 cells (Hsieh et al., 2022). Furthermore, PPARγ, as a nuclear receptor, negatively regulates NF-κB activity by binding to p65 or promoting its ubiquitination and degradation. Ethanol significantly inhibited PPARγ mRNA and protein expression in GES-1 cells while activating p65 transcription and translation. This leads to initiation and amplification of inflammatory responses. The extract from the whole plant of *Physochlaina physaloides* (L.) G.Don (Solanaceae) (BPP) restored its inhibitory effect on NF-κB by upregulating PPARγ (Li et al., 2025).

Notably, NF-κB activation not only initiates transcription of pro-inflammatory cytokines but also upregulates expression of inflammasome components such as NLRP3 and pro-IL-1β. This constitutes the molecular basis for signal transmission from NF-κB to NLRP3 (Liao Y. et al., 2025; Zhou C. et al., 2024). Studies have found that dehydroevodiamine, from the dried fruits of *T. ruticarpum* (A.Juss.) T.G.Hartley (Rutaceae) as a novel ATP-competitive IKKβ inhibitor, not only blocks upstream NF-κB signaling but also downregulates NLRP3 gene transcription. It also blocks the interaction between NLRP3 and downstream factors including ASC and Caspase-1, inhibiting inflammasome assembly. This ultimately reduces indomethacin-induced pro-inflammatory cytokine release in GES-1 cells (Hu et al., 2024). This study confirms that TCM can target the NF-κB/NLRP3 signaling axis, simultaneously blocking both upstream and downstream transmission of inflammatory signals, thereby intercepting the inflammatory cascade.

The NLRP3 inflammasome serves as the downstream executor of the inflammatory cascade. Upon activation, NLRP3 recruits ASC and pro-Caspase-1 to form a complex. On one hand, activated Caspase-1 cleaves gasdermin D (GSDMD) into the N-terminal fragment (N-GSDMD), promoting cell membrane pore formation and triggering pyroptosis. On the other hand, it processes pro-IL-1β into mature IL-1β, further amplifying the inflammatory response (Shi et al., 2015). In ethanol-induced GES-1 cells, chicory-derived scFOS significantly inhibited the protein expression of NLRP3, ASC, and Caspase-1, downregulating cellular inflammatory cytokine levels (Chen et al., 2024). The extract from the whole insect of *Periplaneta americana* L. (Blattidae) (PAE) similarly alleviated ethanol-induced inflammatory injury through inhibition of the NLRP3/Caspase-1 pathway (Fu et al., 2021). Vitexin, an active metabolite from *Crataegus pinnatifida* Bunge (Rosaceae), dose-dependently reduced the protein levels of NLRP3, cleaved-Caspase-1, and IL-1β in LPS plus ATP-treated GES-1 cells, further validating the inhibitory effect of TCM active metabolites on this pathway (Liu J. et al., 2025). Studies on polysaccharide from the dried pericarp of *C. reticulata* Blanco (Rutaceae) involved regulation at both oxidative and inflammatory levels. Ethanol stimulation upregulated the protein levels of NLRP3, ASC, and cleaved-Caspase-1 and increased ROS production in GES-1 cells, while decreasing Nrf2 and HO-1 expression. Pretreatment with *C. reticulata* polysaccharide attenuated these effects. This suggests that its anti-inflammatory mechanism may partly derive from alleviation of oxidative stress—namely, through activation of Nrf2/HO-1 to reduce ROS, thereby weakening ROS-mediated activation signals to the NLRP3 inflammasome (Liang et al., 2025). These studies indicate that TCM can intervene simultaneously at both the NF-κB and NLRP3 levels. This forms a dual-layer anti-inflammatory strategy of inhibiting upstream transcriptional initiation and blocking downstream effector amplification. Some active metabolites may also indirectly inhibit NLRP3 activation by alleviating upstream oxidative stress, reflecting synergistic intervention between oxidative and inflammatory pathways (Figure 3).

**FIGURE 3:**
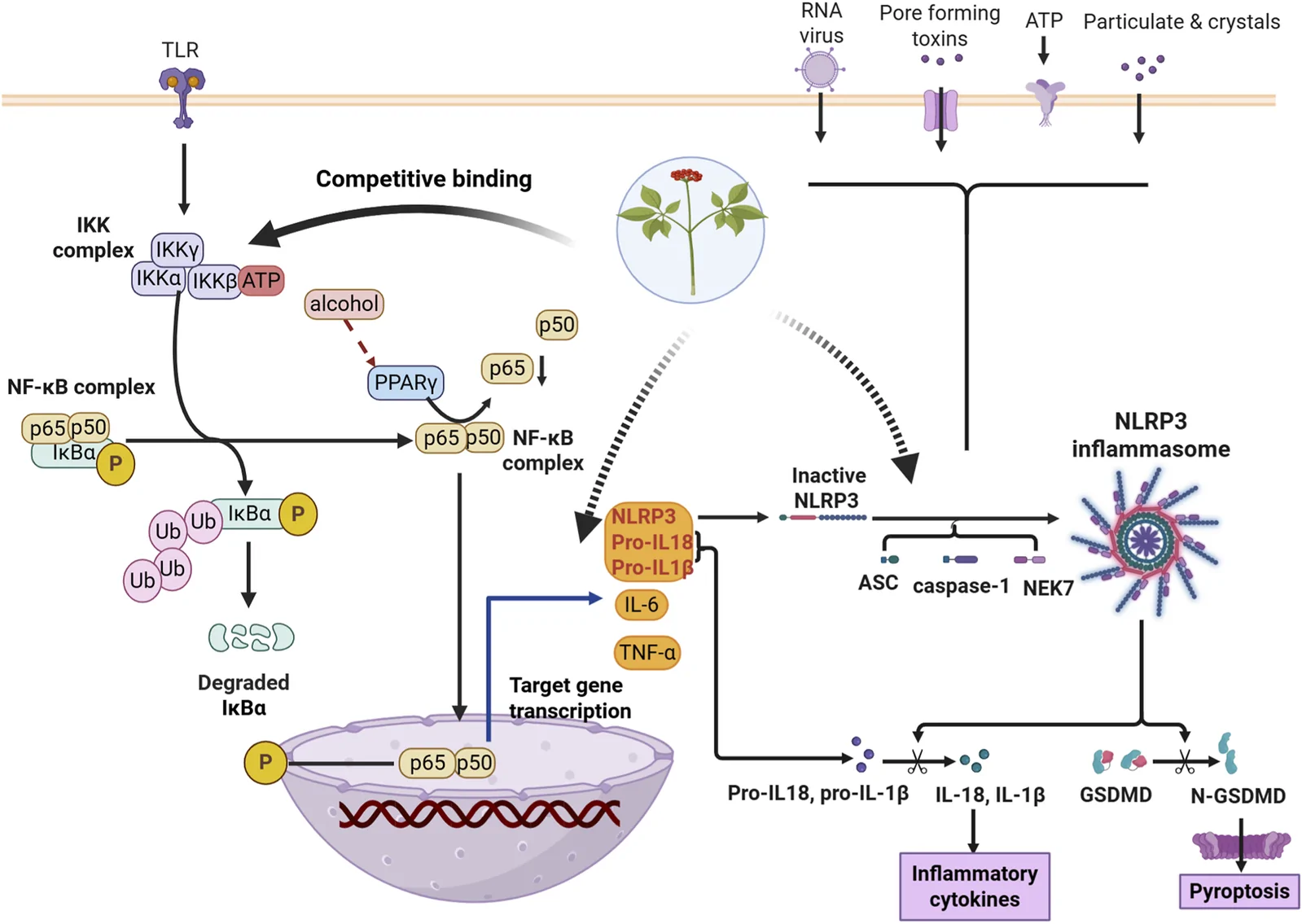
TCM intervenes the inflammatory response of gastric mucosal cells by modulating the NF-κB and NLRP3 pathways. Created in BioRender. Zhang et al. (2026) https://BioRender.com/p3stked.

However, GES-1 monolayer cells lack the participation of immune cell paracrine signals. This potentially underestimates the amplification effect of epithelial–immune interactions on inflammatory cascades in the *in vivo* inflammatory microenvironment. To address this limitation, some studies used GES-1 cells to evaluate the direct protective effects of drugs while simultaneously validating anti-inflammatory mechanisms in RAW264.7 macrophages. For example, in ethanol-induced GES-1 cells, Jianwei Yuyang Tablets (JWYY; composed of *Bupleurum chinense* DC. [Apiaceae], *C. pilosula* (Franch.) Nannf. [Campanulaceae], *P. lactiflora* Pall. [Paeoniaceae], *Corydalis yanhusuo* (Y.H.Chou and Chun C.Hsu) W.T.Wang ex Z.Y.Su and C.Y.Wu [Papaveraceae], *B. striata* (Thunb.) Rchb.f. [Orchidaceae], *Margaritifera* spp. [Margaritiferidae], *Baphicacanthus cusia* (Nees) Bremek. [Acanthaceae], and *G. uralensis* Fisch. ex DC. [Fabaceae]) significantly increased cell viability and maintained cell integrity (Ahmed et al., 2025). Their anti-inflammatory mechanism involving NF-κB pathway inhibition was confirmed using the RAW264.7 macrophage model. This research model suggests that the anti-inflammatory effects of TCM observed in GES-1 monolayer cells need to be further validated in environments containing immune cell interactions. This would more comprehensively assess their actual effects in the *in vivo* inflammatory microenvironment.

### 4.3 Regulation of cell survival and apoptosis

When inflammatory signals persist and cannot be effectively cleared, cells may either survive or undergo death. The dynamic balance between survival and apoptosis of gastric mucosal epithelial cells is crucial for maintaining gastric mucosal integrity and delaying disease progression. The PI3K/AKT pathway is a core signaling axis regulating apoptosis and survival. Upon stimulation by upstream factors such as growth factors, PI3K is activated and generates phosphatidylinositol-3, 4, 5-trisphosphate (PIP3). PIP3 recruits AKT to the cell membrane and promotes its phosphorylation and activation. Activated AKT regulates cell fate directly or indirectly through phosphorylation of downstream target proteins (Xu J. et al., 2023).

In GES-1 cells, various TCM active metabolites exert anti-apoptotic and pro-survival effects by activating this pathway. They regulate the expression of different downstream factors including GSK3β, Bcl-2 family members, and CREB. GSK3β, as a key downstream target of AKT in apoptosis regulation, upon activation phosphorylates the pro-apoptotic protein Bax and promotes its translocation to mitochondria. This induces cytochrome c release and caspase cascade activation, leading to apoptosis (Gupta et al., 2021). Cinnamaldehyde from the stem bark of *N. cassia* (L.) Kosterm. (Lauraceae), by activating the PI3K/AKT pathway, promotes phosphorylation and inactivation of downstream GSK3β. This blocks its pro-apoptotic signals and inhibits aspirin-induced apoptosis (Yan et al., 2024). Additionally, activated AKT can directly regulate the expression of Bcl-2 family proteins, thereby inhibiting apoptosis. The pectic polysaccharide WGPP-1a from the peel of *Benincasa hispida* (Thunb.) Cogn. (Cucurbitaceae), by activating the PI3K/AKT pathway, increased the Bcl-2/Bax ratio and inhibited the expression of the pro-apoptotic proteins Bax and Caspase-3 (Luo et al., 2025). This enhanced cell viability and reduced apoptosis in ethanol-induced GES-1 cells. CREB (cAMP response element-binding protein), upon phosphorylation and activation by AKT, enhances the transcription of anti-apoptotic proteins including Bcl-2, further elevating the cellular survival threshold. Daidzein from the seeds of *Glycine max* (L.) Merr. (Fabaceae), by activating the PI3K/AKT/CREB pathway, upregulated Bcl-2 through CREB-mediated transcription and downregulated Caspase-3 and Bax. This promoted cell survival under stress conditions such as hypoxia and inflammation (Jiang et al., 2025).

In addition to PI3K/AKT-mediated survival signaling, NF-κB also plays an important role in apoptosis regulation. In Section 4.2, NF-κB was described as the core transcriptional hub for inflammatory signals, initiating pro-inflammatory cytokine transcription. However, in the context of cell survival and apoptosis, activated NF-κB can also inhibit apoptosis through regulation of various anti-apoptotic genes. These downstream target genes mainly include Bcl-2 family anti-apoptotic members (e.g., Bcl-2, Bcl-xL) and inhibitors of apoptosis protein (IAP) family members (e.g., c-IAP1/2, XIAP). These block apoptotic signal transmission at the mitochondrial damage level and the caspase execution level, respectively (Foo and Nolan, 1999). In GES-1 cells, the peptide from *Tricholoma matsutake* (S.Ito and S.Imai) Singer (Tricholomataceae) pretreatment inhibited ethanol-induced apoptosis by suppressing NF-κB phosphorylation and nuclear translocation, downregulating Bax and upregulating Bcl-2 expression (Li et al., 2021). Furthermore, at the level of pyroptosis, ginsenoside Rg1 from the roots of *Panax ginseng* C.A.Mey. (Araliaceae) inhibited nigericin-induced GES-1 cell pyroptosis through regulation of the NF-κB/NLRP3/GSDMD pathway (Zhou Z. et al., 2024). This further expands the functional spectrum of NF-κB in programmed cell death regulation.

Similar to NF-κB, the MAPK family was described in Section 4.1 as an upstream regulator of Nrf2, participating in oxidative stress signal transduction. However, when the effect shifts toward cell fate regulation, the functions of MAPK subtypes also change. ERK activation is typically associated with cell survival and proliferation, whereas sustained activation of JNK and p38 tends to induce apoptosis. In GES-1 cells, the sterols from the green embryo of *Nelumbo nucifera* Gaertn. (Nelumbonaceae) (LPSs) protected cells from ethanol-induced apoptotic injury by promoting ERK phosphorylation and inhibiting p38/p-JNK phosphorylation. This upregulated the anti-apoptotic protein Bcl-2 and downregulated the pro-apoptotic proteins Bax and Caspase-3 (Zhang et al., 2021). This finding indicates that TCM can differentially regulate MAPK subtypes, maintaining ERK pro-survival signaling while inhibiting JNK/p38 pro-apoptotic signals, thereby determining cell survival fate.

The three distinct pathways—PI3K/AKT, NF-κB, and MAPK—ultimately converge on the expression of Bcl-2 family proteins and Caspase family members in the regulation of cell survival and death. PI3K/AKT maintains the Bcl-2/Bax balance directly or indirectly through regulation of downstream target genes. NF-κB transcriptionally upregulates Bcl-2 and IAP family members. MAPK subtypes differentially regulate apoptosis-related protein expression through differential modulation of ERK and JNK/p38. TCM can exert anti-apoptotic and pro-survival effects through these three pathways, reflecting its multi-pathway regulatory characteristics (Figure 4).

**FIGURE 4:**
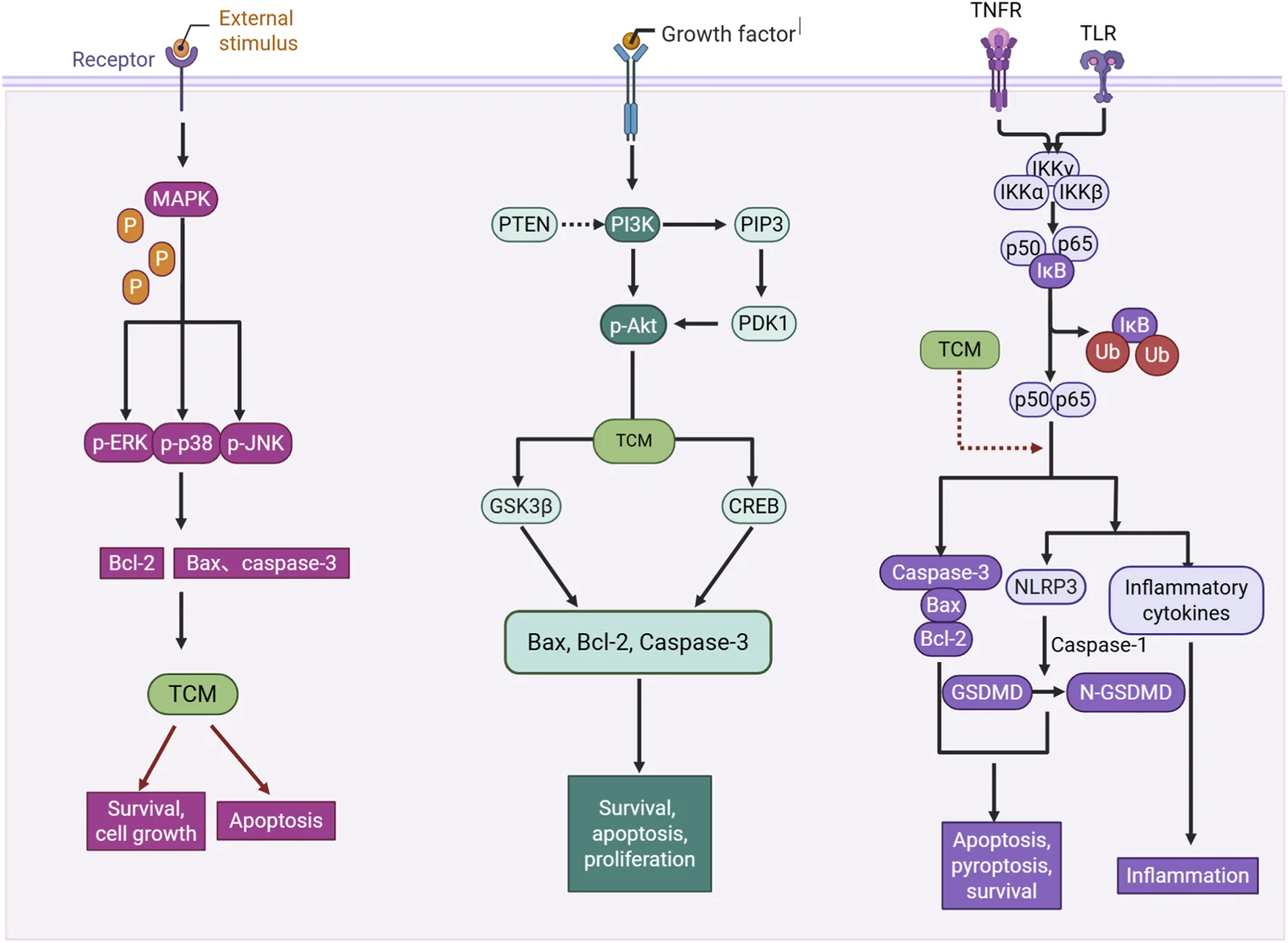
TCM regulates the survival and apoptosis of gastric mucosal cells through the MAPK PI3K/Akt, and NF-κB signaling pathways. Created in BioRender. Zhang et al. (2026) https://BioRender.com/p3stked.

### 4.4 Enhancement of mucosal barrier function

The gastric mucosal barrier is composed of the mucus layer and the epithelial cell layer. Its integrity depends on the coordinated maintenance of tight junctions, adherens junctions, and mucin secretion. When cells successfully resist apoptotic signals and maintain survival, barrier function repair becomes a key step in restoring gastric tissue homeostasis. However, as a monolayer cell model, GES-1 cannot fully simulate the multi-layered barrier structure or cell–cell interactions of the *in vivo* gastric mucosa. Therefore, this section provides only a brief discussion based on existing *in vitro* evidence.

The epidermal growth factor receptor (EGFR) signaling pathway is one of the important pathways regulating gastric mucosal epithelial barrier function. EGF binds to EGFR and promotes its phosphorylation. This in turn phosphorylates and activates downstream extracellular signal-regulated kinase (ERK), generating p-ERK that translocates to the nucleus. p-ERK initiates expression of genes including Cyclin D1, ultimately driving epithelial cell proliferation and promoting structural reconstruction of the damaged barrier (Liu et al., 2023). In ethanol-induced GES-1 cell injury, flavonoids from the roots and rhizomes of *G. uralensis* Fisch. ex DC. (Fabaceae) promoted cell migration and proliferation through activation of the EGFR/ERK pathway (Wu Y. et al., 2023).

Regarding the mucus layer barrier, MUC5AC is the most abundant mucin on the gastric mucosal surface. Its expression level directly determines the thickness and defensive function of the mucus layer. Network pharmacology combined with transcriptomic analysis revealed that Jiawei Lianpu Yin (JWLPY; composed of *C. chinensis* Franch. [Ranunculaceae], *M. officinalis* Rehder and E.H.Wilson [Magnoliaceae], *Pinellia ternata* (Thunb.) Makino [Araceae], *Acorus gramineus* Sol. ex Aiton [Acoraceae], Glycine max (L.) Merr. [Fabaceae], *Phragmites australis* (Cav.) Trin. ex Steud. [Poaceae], *Gardenia jasminoides* J.Ellis [Rubiaceae], *A. macrocephala* Koidz. [Asteraceae], *Tegillarca granosa* (Linnaeus) [Arcidae], *Taraxacum mongolicum* Hand-Mazz. [Asteraceae], and *G. uralensis* Fisch. ex DC. [Fabaceae]) promotes MUC5AC assembly and synthesis in the endoplasmic reticulum through activation of the IRE1-XBP1 signaling pathway. This enhances mucus layer barrier function and inhibits *H. pylori* colonization in the gastric mucosa (Zhang et al., 2025). This study validated pathway-related gene and protein changes in an *H. pylori*-infected gastritis rat model. It further confirmed the dependence of MUC5AC on the IRE1-XBP1 pathway in GES-1 cells using the IRE1 inhibitor APY. This demonstrates a complete evidence chain for TCM gastric mucosal protection mechanisms from *in vivo* to *in vitro*.

It should be noted that although mucin synthesis and tight junction assembly can be partially assessed in GES-1 cells, tissue-level functional validation of barrier function still requires animal models or organoid models.

### 4.5 Improvement of mitochondrial function and energy metabolism

Gastric mucosal epithelial cells require substantial energy supply and protein synthesis to maintain survival, proliferation, and barrier repair functions. This process is highly dependent on mitochondrial oxidative phosphorylation and endoplasmic reticulum protein folding processing, which are coupled through calcium signaling. When cells are stimulated by acute damaging factors such as ethanol, calcium ions are released excessively from the endoplasmic reticulum into the cytoplasm. This causes depletion of the endoplasmic reticulum calcium store, severely interfering with its protein folding function and directly inducing endoplasmic reticulum stress (Gadet et al., 2024). Simultaneously, the abnormally elevated cytoplasmic calcium ions are excessively taken up by mitochondria, leading to mitochondrial calcium overload. This disrupts oxidative phosphorylation and reduces energy synthesis. Energy deficiency further exacerbates the protein folding burden on the endoplasmic reticulum (Lim et al., 2023). Based on these mechanisms, mitochondrial dysfunction-induced energy crisis and endoplasmic reticulum stress-triggered unfolded protein response (UPR) often occur simultaneously. They mutually reinforce to form a vicious cycle that ultimately leads to cellular energy depletion and apoptosis (Fan and Simmen, 2019). In GES-1 cells, TCM can break this cycle by repairing energy metabolism, alleviating endoplasmic reticulum stress, and blocking the vicious connection between them, thereby maintaining cellular energy metabolic homeostasis.

In terms of repairing energy metabolism, the nicotinamide metabolic pathway and mitochondrial dynamics are two key links in maintaining cellular ATP supply. Nicotinamide is a critical precursor for NAD^+^/NADH synthesis. NAD^+^/NADH is a core coenzyme of the electron transport chain in mitochondrial oxidative phosphorylation, directly determining ATP production rate (Tannous et al., 2021). The polysaccharide from *Dendrobium huoshanense* C.Z.Tang and S.J.Cheng (Orchidaceae) restored NAD^+^/NADH balance in damaged GES-1 cells through regulation of nicotinamide metabolism, thereby ensuring energy supply and cell survival (Xie et al., 2023). As the cell’s energy factory, normal mitochondrial function is the structural basis for efficient energy metabolism. The dynamic balance between fusion and fission determines mitochondrial network integrity and respiratory chain efficiency. In GES-1 cells, the sea cucumber peptide VLLY from *Apostichopus japonicus* (Selenka, 1867) (Stichopodidae) inhibited ethanol-induced excessive mitochondrial fission by downregulating fission proteins Drp1/Fis1 and upregulating fusion protein Mfn2. This restored mitochondrial respiratory chain function and ATP synthesis capacity (Zheng et al., 2023).

The endoplasmic reticulum is a key site for protein synthesis, folding, and processing. When damaged factors lead to accumulation of unfolded or misfolded proteins in the endoplasmic reticulum, endoplasmic reticulum stress is triggered and the UPR is initiated. Moderate UPR is protective, but sustained and excessive stress initiates apoptotic programs through specific pathways (Gawlak-Socka et al., 2026). The PERK/eIF2α/CHOP pathway is one of the key signaling axes mediating the transition from endoplasmic reticulum stress to apoptosis. PERK (protein kinase R-like endoplasmic reticulum kinase) is one of three UPR sensors on the endoplasmic reticulum membrane. Upon accumulation of unfolded proteins, activated PERK phosphorylates eIF2α (eukaryotic translation initiation factor 2α subunit). This selectively upregulates ATF4 translation, subsequently activating the pro-apoptotic factor CHOP expression and ultimately initiating apoptosis (Wang Z. et al., 2024). Active metabolites from the resinous wood of *Aquilaria sinensis* (Lour.) Spreng. (Thymelaeaceae) inhibited the PERK/eIF2α/CHOP pathway by downregulating the phosphorylation levels of PERK and eIF2α and the protein expression of CHOP. This blocked sustained endoplasmic reticulum stress activation induced by taurocholic acid in GES-1 cells (Zhang et al., 2023; Ma J. et al., 2022).

IP3R (inositol 1, 4, 5-trisphosphate receptor) is a calcium release channel on the endoplasmic reticulum membrane. Its excessive activation is a key node for transmitting calcium overload signals from the endoplasmic reticulum to mitochondria. Dan-Shen-Yin (DSY; composed of *Salvia miltiorrhiza* Bunge [Lamiaceae], *A. villosum Lour.* [Zingiberaceae], and *Santalum album* L. [Santalaceae]) exerted protective effects in ethanol-induced GES-1 cells by inhibiting excessive IP3R activation, preventing abnormal calcium release from the endoplasmic reticulum into the cytoplasm. It also preserved endoplasmic reticulum calcium homeostasis and mitochondrial energy metabolism function (Zhou et al., 2025).

These studies reveal multiple pathways through which TCM maintains energy metabolic homeostasis in acutely injured GES-1 cells. However, most studies rely on indirect indicators such as metabolomics and protein expression to infer energy status. They lack direct measurements of ATP synthesis rate or oxygen consumption rate. This highlights a common technical challenge in TCM serum pharmacology: serum proteins, pigments, and redox/fluorescent components can interfere with ATP, mitochondrial membrane potential, and ROS readouts. Therefore, when directly assessing mitochondrial function in TCM studies, blank or vehicle serum, heat-inactivated or dialyzed serum, and medium controls should be included, together with orthogonal validation such as mitochondrial complex activity assays or genetically encoded ATP sensors.

### 4.6 Model extension for chronic stimulation and precancerous molecular events

Unlike the acute factor-induced transient injury models discussed above, some studies used long-term, repeated stimulation of GES-1 cells with MNNG, deoxycholic acid, or *H. pylori* to obtain irreversibly malignant gastric precancerous lesion cell models (MC cells). However, these remain *in vitro* surrogate platforms based on monolayer immortalized cell lines and cannot fully simulate tissue-level pathological progression. The studies reviewed in this section should be understood as preliminary screening evidence for TCM intervention in early precancerous molecular events. Their conclusions require further validation in organoid or animal models.

#### 4.6.1 Regulation of cell proliferation, apoptosis, and EMT

STAT3 (signal transducer and activator of transcription 3) is a key oncogene. Its excessive activation is a critical event in the transition from chronic inflammation to gastric cancer (Ouyang et al., 2022). Under physiological conditions, STAT3 activation in normal gastric mucosal epithelial cells is rapid and transient, tightly regulated by negative feedback. However, under sustained stimulation by chronic factors including *H. pylori*, bile acids, and chemical carcinogens, cytokines such as interleukin-6 (IL-6) in the inflammatory microenvironment continuously stimulate gastric mucosal epithelial cells. Upon binding of IL-6 to its receptor, Janus kinase (JAK) family members are phosphorylated and activated. They subsequently recruit and phosphorylate STAT3 at tyrosine residues. Phosphorylated STAT3 forms homodimers, translocates to the nucleus, and binds to DNA response elements in the promoter regions of target genes. This initiates transcription of downstream genes (Fan et al., 2013), driving abnormal cell proliferation, apoptosis resistance, and EMT, providing key targets for TCM intervention in malignant transformation.

Eliminating abnormal cells and restoring normal apoptosis–proliferation balance is an important strategy for blocking malignant transformation. Asarinin from the roots and rhizomes of *Asarum sieboldii* Miq. (Aristolochiaceae), in MNNG-induced malignantly transformed GES-1 cells, downregulated STAT3 phosphorylation levels and its downstream target proteins Bcl-2 and cyclin D1. This demonstrated significant proliferation inhibition and apoptosis induction effects. The STAT3 activator colivelin effectively attenuated these downregulatory effects and inhibited asarinin-induced apoptosis, confirming that this effect depends on inhibition of the STAT3 signaling axis (Zhao M. et al., 2024). Notably, asarinin showed no obvious cytotoxicity in normal GES-1 cells, suggesting its selective action on STAT3-dependent malignantly transformed cells.

EMT is the process by which epithelial cells transform into a mesenchymal phenotype. Its activation is the basis for cells to acquire migratory and invasive capabilities. Phosphorylated STAT3 can directly enhance the transcription of core EMT transcription factors including Snail. It also binds to enhancer elements in the Vimentin gene promoter region to activate its expression, promoting epithelial–mesenchymal transition (Sadrkhanloo et al., 2022). In a chenodeoxycholic acid-induced GES-1 gastric precancerous lesion model, Yiqi Huayu Jiedu Decoction (YQHYJD; composed of *Astragalus mongholicus* Bunge [Fabaceae], *C. pilosula* (Franch.) Nannf. [Campanulaceae], *Panax notoginseng* (Burkill) F.H.Chen [Araliaceae], *A. macrocephala* Koidz. [Asteraceae], *C. reticulata* Blanco [Rutaceae], *A. villosum Lour.* [Zingiberaceae], *T. mongolicum* Hand.-Mazz. [Asteraceae], and *G. uralensis* Fisch. ex DC. [Fabaceae]) intervention effectively inhibited JAK2/STAT3 activation. This was accompanied by downregulation of Snail, Vimentin, and other EMT markers and reversal of the partial EMT (pEMT) phenotype. This confirmed that YQHYJD blocks gastric inflammation-to-carcinoma transition through inhibition of JAK2/STAT3-mediated pEMT (Yu W. et al., 2025). Another study found that the MNNG-GES-1 cell line generated by treating GES-1 cells with MNNG exhibited enhanced proliferation, migration, and invasion capabilities. Wendan Decoction (WDD; composed of *P. ternata* (Thunb.) Makino [Araceae], *Bambusa tuldoides* Munro [Poaceae], *C. aurantium* L. [Rutaceae], *C. reticulata* Blanco [Rutaceae], Wolfiporia cocos (F.A.Wolf) Ryvarden and Gilb. [Polyporaceae], Zingiber officinale Rosc. [Zingiberaceae], *Z. jujuba* Mill. [Rhamnaceae], and *G. uralensis* Fisch. ex DC. [Fabaceae]) inhibited these malignant tumor-related phenotypes while reducing the protein expression levels of p-JAK2 and p-STAT3. This suggests that its inhibition of EMT may be related to regulation of JAK2/STAT3 phosphorylation (Zhang et al., 2026). However, this study did not directly detect changes in EMT markers. The STAT3 downstream EMT regulatory links in this model require further validation to complete the mechanistic understanding of its blockade of inflammation-to-carcinoma transition.

In addition to the STAT3/Snail axis, the JNK/c-Jun/Slug axis has also been confirmed to play a key role in EMT regulation of gastric mucosal epithelial cells. As an important member of the Snail family, Slug initiates epithelial–mesenchymal transition through transcriptional repression of E-cadherin and activation of N-cadherin and Vimentin (Brabletz et al., 2021). Its upstream signal, JNK—a stress-responsive kinase of the MAPK family—phosphorylates and activates c-Jun under inflammatory or oxidative stress. This further drives Slug transcription (Morgan et al., 2021). In an MNNG-induced PLGC rat model and GES-1 malignant transformation cell model, Huayu Xiaopi Decoction (HYXPT; composed of *P. ginseng* C.A.Mey. [Araliaceae], *Z. officinale* Rosc. [Zingiberaceae], *A. macrocephala* Koidz. [Asteraceae], *G. uralensis* Fisch. ex DC. [Fabaceae], *C. aurantium* L. [Rutaceae], *C. chinensis* Franch. [Ranunculaceae], *P. ternata* (Thunb.) Makino [Araceae], *Wolfiporia cocos* (F.A.Wolf) Ryvarden and Gilb. [Polyporaceae], *C. reticulata* Blanco [Rutaceae], *M. officinalis* Rehder and E.H.Wilson [Magnoliaceae], *D. costus* Falc. [Asteraceae], *A. villosum Lour.* [Zingiberaceae], *Curcuma phaeocaulis Valeton* [Zingiberaceae], *P. notoginseng* (Burkill) F.H.Chen [Araliaceae], *Daemonorops draco* (Willd.) Blume [Arecaceae], and *Buthus martensii* Karsch [Buthidae]) inhibited JNK and c-Jun phosphorylation in a dose-dependent manner. It reduced Slug expression and reversed EMT biomarker expression, thereby blocking the EMT process (Chen et al., 2026). These studies respectively target the STAT3/Snail and JNK/c-Jun/Slug signaling axes, converging from different upstream signaling nodes on the final execution stage of EMT.

#### 4.6.2 Abnormal differentiation

Beyond acquiring proliferation, apoptosis resistance, and migration/invasion capabilities, malignantly transformed cells also exhibit abnormal differentiation direction. Particularly, the shift of gastric mucosal epithelial cells toward an intestinal epithelial phenotype (intestinal metaplasia) is another key node in the transition from gastric inflammation to carcinoma. NF-κB signaling plays an important role in driving gastric mucosal epithelial cells toward intestinal metaplasia. CDX2 is a specific marker of the intestinal phenotype. Its high expression is closely associated with abnormal gastric intestinal differentiation. Sustained activation of the NF-κB pathway promotes CDX2 transcription and intestinal differentiation. Under sustained stimulation by damaging factors, p65 is activated and translocates to the nucleus. It directly binds to specific DNA sequences in the CDX2 gene promoter region to drive its transcription (Li et al., 2019). In MNNG plus TNF-α co-stimulated GES-1 cells, Weifuchun (WFC; composed of *P. ginseng* C.A.Mey. [Araliaceae], *Isodon amethystoides* (Benth.) H.Hara [Lamiaceae], and *C. aurantium L*. [Rutaceae]) inhibited the aberrant transcription of CDX2 and blocked the initiation of the intestinal metaplasia program by inhibiting p65 nuclear translocation and p65 binding to the CDX2 promoter (Xie D. et al., 2024). Furthermore, in a GIM cell model established by inducing GES-1 cells with deoxycholic acid, Weiyan Tongluo Granules (WYTLG; composed of *A. mongholicus* Bunge [Fabaceae], *Ficus simplicissima Lour.* [Moraceae], *A. macrocephala* Koidz. [Asteraceae], Scutellaria barbata D.Don [Lamiaceae], Curcuma phaeocaulis Valeton [Zingiberaceae], *Sparganium stoloniferum* Buch.-Ham. [Sparganiaceae], *Margaritifera* spp. [Margaritiferidae], and *C. aurantium* L. [Rutaceae]) reduced the phosphorylation level of p65 in the nucleus and CDX2 protein expression. This indicates regulation of CDX2-driven intestinal differentiation through the NF-κB signaling pathway (Liang et al., 2026).

These findings suggest that TCM can intervene in STAT3/JAK-mediated proliferation, apoptosis, and EMT, as well as NF-κB/CDX2-driven intestinal metaplasia in GES-1 malignant transformation models. This attenuates the malignant phenotypes of MC cells at the monolayer cell level. However, the above evidence all originates from immortalized cell lines. Tissue-level functional confirmation of glandular reconstruction and intestinal metaplasia reversal requires organoid validation.

## 5 TCM intervention mechanisms based on gastric organoid models

Unlike GES-1 monolayer models that focus on molecular events in a single cell type, gastric organoids—as *in vitro* models that closely simulate the physiological structure and function of *in vivo* gastric tissue—not only retain the genetic background and pathological features of donor tissues but also three-dimensionally recapitulate gastric stem cell self-renewal, differentiation lineages, and mucosal barrier function. In gastric mucosal inflammation-to-carcinoma transition research, the core value of gastric organoids lies in constructing a continuous pathological spectrum from chronic inflammation to gastric precancerous lesions and then to gastric cancer. This provides tissue-level biological interpretation for validating molecular mechanisms discovered in GES-1 cell models. It also enables evaluation of the clinical translation potential of TCM in patient-derived pathological models.

The gastric organoid studies reviewed in this section provide higher-tier evidence for tissue-level functional validation (see Table 1 for the complete evidence-level classification of each study). Patient-derived organoids are classified as the highest evidence level in our framework, while induced organoids represent an intermediate tier.

**TABLE 1:** Mechanisms of TCM interventions in gastric inflammation-to-carcinoma transition: summary of *in vitro* models, signaling pathways, and evidence levels.

Biological function	TCM	Model system	Intervention node	Drug and concentration	Groups	Pathways/Targets	*In vitro* evidence level	Reliability and limitations	Ref.
Antioxidative stress	Polysaccharide extract from *Amomum villosum Lour* (AVLP-2)	*In vitro:* GES-1, ethanol/LPS; *In vivo:* Rats, ethanol-induced acute injury	Acute mucosal injury	Extract; *In vitro:* 1–10 μg/mL, 12 h; *In vivo:* 2–4 mg/kg (duration unclear)	Model, AVLP-2, LY294002	Increase: SOD Decrease: MDA, iNOS	C	Reliability: Structural characterization complete; PI3K pathway validated with inhibitor. Limitations: *In vivo* administration duration not clearly specified; no *in vivo* pathway inhibition validation	(Liu, H. et al., 2022)
	Costunolide	*In vitro:* GES-1, MNNG 70 μM, 24 h; *In vivo:* MNNG + ethanol + irregular diet-induced CAG, 10 weeks	Chronic atrophic gastritis	Isolated compound; *In vitro:* 2.5–10 μM, 2 h; *In vivo:* 0–20 mg/kg, 4 weeks	Model, Costunolide, vitamin, Nrf2 inhibitor	Increase: SOD, GSH, HO-1 Decrease: ROS, MDA	C	Reliability: Clear Nrf2 targeting; Limitations: No Nrf2 knockout animal validation	(Wang, R. et al., 2024)
	Xiangshao Decoction (XST)	*In vitro*: GES-1, H_2_O_2_ 400 μM, 2 h; *In vivo*: Rats, acetic acid 20%-induced chronic gastric ulcer, 14 days postsurgery	Acute mucosal injury	Extract; *In vivo*: 500–2000 mg/100g, 14 days; *In vitro*: 100–400 μg/mL, 24 h	Model, XST, omeprazole, Nrf2 siRNA	Increase: Nrf2, NQO1, HO-1, SOD2 Decrease: Keap1, ROS, MDA, IL-1β, IL-6, TNF-α	C	Reliability: Clear Nrf2 targeting, improves neuroinflammation; Limitations: Model etiology differs from clinical	(Gong, H. et al., 2023)
	Short-chain fructo-oligosaccharides (scFOS)	*In vitro*: GES-1, ethanol 7%, 2 h (no *in vivo*)	Acute mucosal injury	Isolated compound; 50–100 μg/mL, 24 h	Model, scFOS	Increase: Nrf2, HO-1, SOD1, SOD2 Decrease: Keap1, ROS	C	Reliability: First report of protective effect; Limitations: *In vitro* only, DP-activity relationship needs further study	(Chen, Y. et al., 2024)
	Piperine	*In vitro*: GES-1, ethanol 1.0 M, 2 h; *In vivo*: Rats, absolute ethanol 4 mL/kg, single dose	Acute mucosal injury	Isolated compound; *In vivo*: 25–100 mg/kg, 7 days; *In vitro*: 5–20 mg/L, 24 h	Model, piperine, lansoprazole	Modulates Nrf2/HO-1 and MAPK signaling Increase: SOD, CAT, GSH Decrease: Keap1, JNK, p38	C	Reliability: Consistent *in vitro*/*in vivo*, Nrf2/MAPK validation; Limitations: No pathway inhibitor validation	(Duan, Z. et al., 2022)
Anti-inflammation	Dangshen Huangjiu	*In vitro*: GES-1, ethanol 2%, 24 h; *In vivo*: MNNG 0.04 M + fasting/feeding-induced, 10 weeks	Acute mucosal injury	Extract; *In vivo*: 10–20 mL/kg, 4 weeks; *In vitro*: 25–100 μg/mL, 24 h	Model, Dangshen Huangjiu, hydrotalcite	Inhibits NF-κB signaling Decrease: p-Akt, NF-κB-p65, p-NF-κB-p65, TNF-α, IL-6, IL-1β	C	Reliability: Validates Akt/NF-κB pathway; Limitations: Alcohol content may interfere	(Xu, Q. et al., 2023)
	Chinese Yam aqueous extract (CYW)	*In vitro*: GES-1 + RAW264.7 co-culture, ethanol 10%, 3 h; *In vivo*: Rats, absolute ethanol 10 mL/kg, single dose	Acute mucosal injury	Extract; *In vivo*: 0.31–3.14 g/kg, 14 days; *In vitro*: 0.1–0.4 mg/mL, 24 h	Model, CYW, omeprazole	Decrease: p-NF-κB, p-IκB-α, TNF-α, IL-6, IL-1β	C	Reliability: HPLC-QTOF-MS + GNPS molecular networking; Limitations: No pathway inhibitor validation	(Xie, Y. et al., 2024)
	Quercetin	*In vitro*: GES-1, TNF-α 30 ng/mL (no *in vivo*)	Acute mucosal injury	Isolated compound; 0.1–1 μM, 1 h	Control, TNF-α, quercetin + inhibitors	Inhibits TNFR-c-Src-ERK1/2-NF-κB pathway Decrease: MMP-9	C	Reliability: Complete signaling pathway validation; Limitations: *In vitro* only, no animal study	(Hsieh, H. L. et al., 2022)
	*Physochlaina physaloides* (L) G. Don. (BPP)	*In vitro*: GES-1, ethanol 6%, 6 h; *In vivo*: Rats, 56% absolute ethanol-induced, 4 weeks	Acute mucosal injury	Extract; *In vivo*: 1.28–11.53 mg/kg, 2 weeks; *In vitro*: 50–400 μg/mL, 24 h	Model, BPP, ranitidine/teprenone	Increase: PPARα, PPARγ, CPT-1, FABP3	C	Reliability: Multi-omics integration, PPAR/NF-κB pathway; Limitations: Active compounds have potential side effects	(Li, S. et al., 2025)
	Short-chain fructo-oligosaccharides (scFOS)	*In vitro*: GES-1, ethanol 7%, 2 h (no *in vivo*)	Acute mucosal injury	Isolated compound; 50–100 μg/mL, 24 h	Model, scFOS	Decrease: NLRP3, ASC, Caspase-1, IL-1β, TNF-α, IL-6, iNOS, NO	C	Reliability: First report of protective effect; Limitations: *In vitro* only, DP-activity relationship needs further study	(Chen, Y. et al., 2024)
	*Periplaneta americana L.* Extract (PAE)	*In vitro*: GES-1, ethanol 100 μM; *In vivo*: Mice, absolute ethanol 6 mL/kg, single dose	Acute mucosal injury	Extract; *In vivo*: 200–800 mg/kg, 3 days; *In vitro*: 5–80 μg/mL, 24 h	Model, PAE, sucralfate	Inhibits NLRP3/Caspase-1 pathway	C	Reliability: Active fraction screened; Limitations: Mechanism study relatively superficial	(Fu, S. et al., 2021)
​	Dehydroevodiamine	*In vitro*: GES-1, indomethacin (IDO) 300 μM, 24 h; *In vivo*: Rats, IDO 5 mg/kg, 5 days	Acute mucosal injury	Isolated compound; *In vitro*: 2.5–5 μM, 24 h; *In vivo*: 5–10 mg/kg, 5 days	Model, DHE, omeprazole, IKKβ activator	Inhibits IKKβ-p65/NLRP3 pathway Increase: Bcl-2, Bcl-2/Bax Decreased: TNF-α, IL-1β, IL-18, Bax, active Caspase-3	C	Reliability: Clear IKKβ target and binding sites (GLU-149, GLU-49, ASP-103); Limitations: Only GES-1 cells used	(Hu, Q. et al., 2024)
	Vitexin	*In vitro*: GES-1, LPS + ATP; *In vivo*: MNNG 170 μg/mL-induced CAG, 10 weeks	Acute mucosal injury	Isolated compound; *In vivo*: 30 mg/kg, 6 weeks; *In vitro*: 2.5–100 μM, 24 h	Model, Vitexin, folic acid, NLRP3 inhibitor	Decreased: NLRP3, cleaved-Caspase-1, IL-1β	C	Reliability: Direct NLRP3 targeting (CETSA/DARTS/biotin labeling); Limitations: No NLRP3 knockout animal validation	(Liu, J. et al., 2025)
	*Citri Reticulatae* Polysaccharide	*In vitro*: GES-1, ethanol 1 M, 4 h; *In vivo*: Mice, 56% ethanol, 7 days	Acute mucosal injury	Extract; *In vivo*: 50–250 mg/kg, 5 days; *In vitro*: 1 mg/mL, 24 h	Model, *Citri Reticulatae* Polysaccharide low/high dose	Increase: Nrf2, HO-1 Decreased: NLRP3, ASC, cleaved-Caspase-1, ROS	C	Reliability: Nrf2/NLRP3 pathway validation; Limitations: No inhibitor validation	(Liang, H. et al., 2025)
	Jianwei Yuyang Tablets (JWYY)	*In vitro*: GES-1 (ethanol)/RAW264.7 (LPS); *In vivo*: Mice, ethanol 10 mL/kg, single dose	Acute mucosal injury	Extract; *In vivo*: 20–80 mg/kg, 7 days; *In vitro*: 10%–20%, 12 h	Model, JWYY, omeprazole	Inhibit JAK2/STAT3 pathway and NF-κB pathway; activate PI3K/AKT pathway Increase: p-AKT, Bcl-2, ZO-1 Decrease: p-JAK2, p-STAT3, p-p65, Bax, Cleaved-Caspase 3	C	Reliability: RNA-seq + network pharmacology, dual JAK2-STAT3/NF-κB pathways; Limitations: Complex formula, monomers not isolated	(Ahmed, B. et al., 2025)
Regulation of cell survival and apoptosis	Cinnamaldehyde	*In vitro*: GES-1, aspirin/erastin; *In vivo*: Mice, aspirin 300 mg/kg, 14 days	Acute mucosal injury	Isolated compound; *In vitro*: 5–20 μM, 24 h; *In vivo*: 150–500 mg/kg, 15 days	Model, Cinnamaldehyde, PI3K/AKT modulators	Activate PI3K/AKT/mTOR/GSK3β and PI3K/AKT/Nrf2/HO-1 pathway	C	Reliability: Multi-omics reveals autophagy/apoptosis/ferroptosis; Limitations: Only acute injury model	(Yan, S. et al., 2024)
	Wax gourd peel pectic polysaccharide (WGPP-1a)	*In vitro*: GES-1, ethanol 4%, 2 h; *In vivo*: Mice, absolute ethanol 0.2 mL/10g, 1 h	Acute mucosal injury	Extract; *In vitro*: 100–200 μg/mL, 12 h; *In vivo*: 25–50 mg/kg, 4 days	Model, WGPP-1a, omeprazole	Activates PI3K/AKT pathway Increase: Bcl-2/Bax Decreased:Bax, Caspase-3	C	Reliability: Well-characterized structure, PI3K/AKT validation; Limitations: No SAR study	(Luo, B. et al., 2025)
	Daidzein	*In vitro*: GES-1, ethanol 3%, 24 h; *In vivo*: Rats, ethanol 5 mL/kg, single dose	Acute mucosal injury	Isolated compound; *In vivo*: 300–600 mg/kg, 10 days; *In vitro*: 7.5–90 μM, 24 h	Model, Daidzein, omeprazole, ESR1 inhibitor	Activates PI3K/AKT/CREB pathway Increase: Bcl-2 Decreased:Bax, Caspase-3	C	Reliability: Network pharmacology + molecular dynamics; Limitations: No ESR1 knockout validation	(Jiang, T. et al., 2025)
	Tricholoma matsutake-Derived Peptides	*In vitro*: GES-1, ethanol 5%, 6 h; *In vivo*: Mice, absolute ethanol 0.1 mL/10g, single dose	Acute mucosal injury	Isolated compound; *In vivo*: 50 mg/kg, 14 days; *In vitro*: 200 μg/mL, 24 h	Model, peptides, lansoprazole	Inhibits NF-κB pathway Increase: Bcl-2 Decrease: Bax, Cleaved Caspase-3	C	Reliability: Anti-inflammatory and anti-apoptotic mechanisms; Limitations: Gastric stability of peptides unknown	(Li, M. et al., 2021)
	Lotus plumule sterols (LPSs)	*In vitro*: GES-1, ethanol 90 mM, 4 h (no *in vivo*)	Acute mucosal injury	Isolated compound; 9–12 μg/mL, 48 h	Model, LPS, β-sitosterol	Increase: p-ERK, Bcl-2, SOD, CAT, GSH-px Decrease: p-p38, p-JNK, Bax, Caspase-3, MDA	C	Reliability: Clear composition analysis; Limitations: *In vitro* only, no *in vivo* validation	(Zhang, Y. et al., 2021)
Promotion of gastric epithelial cell regeneration and repair	Licorice flavonoids	*In vitro*: GES-1, ethanol 5%, 3 h; *In vivo*: Rats, ethanol 5 mL/kg, repeated (days 1,5,9)	Acute mucosal injury	Isolated compound; *In vivo*: 150–300 mg/kg, 10 days; *In vitro*: 2.5–15 μg/mL, 24 h	Model, Licorice flavonoids, omeprazole	Activates EGFR/ERK pathway	C	Reliability: 16S rDNA + SCFA integration, gut microbiome-mucosal repair axis; Limitations: No germ-free or FMT validation	(Wu, Y. et al., 2023)
	Jiawei Lianpu Yin (JWLPY)	*In vitro:* GES-1, *H. pylori* infection; *In vivo: H. pylori-*infected rats	Chronic active gastritis	Extract; *In vivo:* 0.567–2.268 g/mL, 14 days; *In vitro:* concentration unspecified	Model, JWLPY, PPI/RBC	Activates IRE1-XBP1 signaling Increase: MUC5AC	C	Reliability: First to reveal IRE1/XBP1s-MUC5AC pathway; Limitations: No gene knockout validation	(Zhang, X. et al., 2025)
Improving Mitochondrial Function and Energy Metabolism	Val-Leu-Leu-Tyr (VLLY)	*In vitro*: GES-1, ethanol 4%, 6 h (no *in vivo*)	Acute mucosal injury	Isolated compound; 62.5–250 nmol/mL, 24 h	Model, VLLY, omeprazole, Mdivi-1	Increase: Mfn2, Bcl-XL, Bcl-XL/Bad Decrease: Bax, Bad, Cleaved-Caspase-3, ROS, Drp1, Fis1	C	Reliability: Deep mitochondrial fission mechanism (Fis1/Bax); Limitations: *In vitro* only	(Zheng, Z. et al., 2023)
	Chromone-enriched extract (CPE) from *Aquilaria sinensis*	*In vitro*: GES-1, taurocholic acid (TCA) 1.5 mM, 24 h; *In vivo*: Mice, TCA + ethanol, 5 weeks	Bile reflux gastritis	Extract; *In vitro*: 1–10 μg/mL, 24 h; *In vivo*: 5.68–11.36 g/kg, 4 weeks	Model, CPE, sucralfate	Blocks the PERK-eIF2α-CHOP apoptotic signaling axis Decrease: p-PERK, p-eIF2α, CHOP	C	Reliability: Clear PERK/eIF2α/CHOP pathway and rigorous structural identification of novel compounds; Limitations: Active monomers not identified	(Zhang, H. et al., 2023, Ma, J. et al., 2022)
	Dan-Shen-Yin (DSY)	*In vitro*: GES-1, ethanol (200 mM, 24 h) and ionomycin (2.5 µM, 24 h). *In vivo*: Rat, 56% alcohol-induced (8 g/kg, twice weekly) for 4 weeks	Acute mucosal injury	Extract; *In vitro*: 1–10 µM for 24 h; *In vivo*: 4.536, 9.072, 18.144 g/kg, 7 days	Model, DSY low/medium/high dose, Omeprazole	Inhibited the activation of IP3R-mediated calcium signaling pathway Increase: Bcl-XL, Bcl-2 Decrease: IP3R, GRP75, VDAC1, Caspase-9, Caspase-3, Cytc, Bax	C	Reliability: Multi-omics identified IP3R/calcium pathway; active compounds validated. Limitations: Short *in vivo* treatment; no IP3R knockout/inhibitor validation	(Zhou, Z. et al., 2025)
Chronic stimulation and precancerous molecular events	Asarinin	*In vitro*: MNNG-transformed GES-1 (MC cells), 24 h; *In vivo*: MNU-induced PLGC, 16 weeks	Gastric precancerous lesions	Isolated compound; *In vitro*: 80–140 μM, 24 h; *In vivo*: 5–10 mg/kg, 4 weeks	Model, Asarinin, folic acid, NAC	Decrease: p-STAT3, Bcl-2, cyclin D1	C	Reliability: Targets STAT3 and mitochondrial ROS; Limitations: Potential cytotoxicity concerns	(Zhao, M. et al., 2024)
	Yiqi Huayu Jiedu Decoction (YQHYJD)	*In vitro*: GES-1, DCA 400 μM, 24 h; *In vivo*: DCA + ammonia + irregular diet-induced, 16 weeks	Gastric precancerous lesions	Extract; *In vivo*: 1.079–2.158 mL/100g, 4 weeks; *In vitro*: high/medium dose, 24 h	Model, YQHYJD, AG490, Colivelin	Inhibited JAK2/STAT3 Decrease: Snail, Vimentin	C	Reliability: Validates JAK2/STAT3-mediated pEMT; Limitations: No STAT3 gene knock-out animal	(Yu, W. et al., 2025)
	Wendan Decoction (WDD)	*In vitro*: MC cells (MNNG-transformed); *In vivo*: MNNG multi-factor induced, 10 weeks	Gastric precancerous lesions	Extract; *In vivo*: 1.5–6 g/kg, 4 weeks; *In vitro*: 0.4–1.6 mg/mL, 24 h	Model, WDD, Weifuchun	Decrease: p-JAK2, p-STAT3	C	Reliability: HPLC-Q-TOF-MS identifies 34 components; Limitations: No JAK2/STAT3 gene knockout	(Zhang, X. et al., 2026)
	Huayu Xiaopi Decoction (HYXPT)	*In vitro*: MNNG-transformed GES-1 (MC); *In vivo*: MNNG multi-factor induced, 30 weeks	Gastric precancerous lesions	Extract; *In vivo*: 6.2–24.8 g/kg, 90 days; *In vitro*: 5%–15%, 24 h	Model, HYXPT, folic acid, JNK inhibitor	Inhibited JNK/c-Jun Decrease: Slug, EMT markers	C	Reliability: UHPLC-MS characterization, JNK/c-Jun/Slug pathway; Limitations: Only 44 absorbed components identified	(Chen, H. et al., 2026)
	Weifuchun (WFC)	*In vitro*: GES-1, MNNG 20 µM + TNF-α 50 ng/mL, 12–24 h; *In vivo*: Rat, MNNG (100 mg/L) + ranitidine + irregular diet + hot saline + ethanol (30%), 16–24 weeks	Gastric precancerous lesions	Extract; *In vitro*: 15–120 µg/mL, 2 h; *In vivo*: 1.44 g/kg, 8 weeks (GIM) or 16 weeks (GDys)	Model, WFC, CAPE.	Decrease:CDX2, Ikkβ, i-κbα, p65	C	Reliability: Well-designed *in vivo* models covering both GIM and GDys stages; clear demonstration of WFC inhibiting NF-κB pathway and CDX2 expression; Limitations: Key active components of WFC not identified	(Xie, D. et al., 2024)
​	Weiyan Tongluo Granules (WYTLG)	*In vitro*: GES-1/AGS, DCA 200 μM, 24 h; *In vivo*: MNNG 170 μg/mL + DCA-induced, 15 weeks	Gastric intestinal metaplasia	Extract; *In vivo*: 1.11–4.44 g/kg, 10 weeks; *In vitro*: 5%–20% serum, 48 h	Model, WYTLG, vitamin, PPARγ modulators	Activates PPARγ, and inhibits NF-κB p65 nuclear translocation Decrease: CDX2 expression	C	Reliability: Transcriptomics + clinical microarray validate PPARγ/NF-κB/CDX2 axis; Limitations: No gene knockout animal validation	(Liang, Y. et al., 2026)
Restoration of barrier function	Plumbagin	*In vitro*: MC cells (MNNG-transformed), human organoids; *In vivo*: MNU-induced PLGC, 16 weeks	Gastric precancerous lesions	Isolated compound; *In vivo*: 5–10 mg/kg, 4 weeks; *In vitro*: 5–20 μM, 24 h	Model, Plumbagin, folic acid, ICAM1 modulators	Blocks the ICAM1/ITGB2 signaling axis Increase: ZO-1, occluding, claudin-18 Decrease: MMP7	A	Reliability: Clear ICAM1 target (THR-85), organoid validation; Limitations: Only Asian population samples	(Zhao, M. et al., 2025)
	Paeoniflorin	*In vitro*: GES-1/human gastric organoids, ethanol 3%, 24 h; *In vivo*: Rats, ethanol 5 mL/kg, single dose	Mucosal injury	Isolated compound; *In vivo*: 25–100 mg/kg, 10 days; *In vitro*: 5–10 μM, 24 h	Model, Paeoniflorin, omeprazole/ranitidine/sucralfate	Inhibits PTEN and activates AKT/CREB pathway Increase: p-AKT, p-CREB, TJP1, OCLN, CLDN4, CLDN18	A	Reliability: Clear PTEN target (Pro169), multi-model validation, head-to-head with clinical drugs; Limitations: Low oral bioavailability	(Hu, Q. et al., 2025)
Activation of stem cell regeneration	PEEPA-P5	*In vitro*: ethanol/MNNG induced GES-1, Patient-derived nonatrophic gastritis and chronic atrophic gastritis organoids; *In vivo*: mice, MNNG + ranitidine-induced, 20 weeks	Atrophic gastritis	Isolated compound; *In vitro*: 0.4 μM, 24 h; *In vivo*: 400 μL/day, 2–4 weeks	Model, P5, Stat3 knock-out	Activates EGFR-Stat1and EGFR-ERK signaling Increase: EGFR, ERKs, Stat1	A	Reliability: Direct target EGFR/Stat1, patient-derived organoids; Limitations: Limited *in vivo* stability data	(Li K. et al., 2024)
Reversal of cellular homeostasis imbalance	Luteolin (Lut)	*In vitro*: Patient-derived gastric cancer organoids (PDOs), AGS/BGC823/SGC7901 cells	Gastric cancer	Isolated compound; IC50 27.19 μM (PDOs, 3 days); 29.92–63.06 μM (cells, 24 h)	Control, Lut, carboplatin/norcantharidin	Increase: FOXO3, DUSP1, CDKN1A Decrease:CDX2, KLF4, MUC2, IL1R1, FGFR4	A	Reliability: Patient-derived organoids, comparison with clinical drugs; Limitations: Small sample size (n = 11), no *in vivo* data	(Hao, X. et al., 2023)
	Luteolin (Lut)	*In vitro*: GES-1, CDCA 150 μM, 24 h; Human gastric precancerous organoids (hPCO); *In vivo*: TAM/CDCA-induced models	Gastric precancerous lesions	Isolated compound; *In vivo*: 20–40 mg/kg, 10 days; *In vitro*: 20 μM, 5 days (organoids)	Model, Lut, STAT3 inhibitor, LCN2 antibody	Blocks STAT3/LCN2 pathway Decrease: p-STAT3, LCN2	A	Reliability: Deep mechanism, STAT3 targeting, organoid validation; Limitations: Multi-model validation	(Hao, X. et al., 2025)
	Lianqiao Wendan Decoction (LQWDD)	*In vivo:* Rats, MNNG (200 µg/mL) + 40% ethanol + irregular diet-induced, 40 weeks; *In vitro:* Human GC cell lines; Patient-derived gastric cancer organoids. Clinical: Retrospective analysis of 3 GPL patients treated with LQWDD for 3 months	Gastric cancer	Extract; *In vitro*: Quercetin and kaempferol (40 and 80 μM, 48–72 h); *In vivo*: LQWDD (5.68 and 11.36 g/kg, 26–40 weeks)	*In vitro*: Control, Quercetin/Kaempferol, Oxaliplatin, AMPK-IN-3 groups. *In vivo*: Control, Model, WFC, LQWDD	Activates AMPK pathway Increase: ALDH3A1, NDUFS4 Decrease: ROS	A	Reliability: Proteomics + metabolomics reveal OXPHOS; Limitations: Only 3 clinical cases	(Chi, X. et al., 2025)
	Weifuchun (WFC)	Patient-derived gastric fundic gland polyp organoids	Gastric polyps	Extract; 250 μg/mL, 72 h	Control, WFC, sorafenib, ferroptosis modulators	Increase: SLC7A11 Decrease: ACSL4, ALOX15B	A	Reliability: Patient-derived organoids, ferroptosis induction; Limitations: High inter-individual variability in organoids	(Yue, L. I. et al., 2025)

### 5.1 Restoration of barrier function and blockade of the pro-tumor microenvironment

Sustained damage to the gastric mucosal barrier can activate inflammatory responses and create a pro-tumor microenvironment. This is an important driver of the inflammation-to-carcinoma transition process. Gastric organoids can three-dimensionally recapitulate barrier structures including tight junctions and adherens junctions. This provides a model close to physiological conditions for evaluating drug effects on barrier repair and pro-tumor microenvironment blockade.

Zhao et al. (2025) used MNNG to induce MC cells and performed transcriptomic analysis and single-cell sequencing. They found that the interaction between the cell adhesion molecule ICAM1 and integrin ITGB2 is critical for maintaining intercellular adhesion and gastric mucosal barrier integrity. Plumbagin from the roots of *Plumbago zeylanica* L. (Plumbaginaceae) restored intercellular adhesion integrity by inhibiting ICAM1-ITGB2 complex formation. Its clinical relevance was validated using patient-derived gastric precancerous lesion (GPL) organoids. After plumbagin treatment, GPL organoids exhibited more regular glandular structures and reduced cell shedding and invasive growth. This study conducted molecular mechanism screening in *in vitro* monolayer cells and used gastric organoids to confirm at the histological level the critical significance of barrier function restoration in blocking precancerous lesion progression.

PTEN, as a classic tumor suppressor, maintains the proliferation–apoptosis balance of gastric mucosal epithelial cells through negative regulation of the PI3K/AKT pathway. PTEN expression is abnormally elevated during gastric mucosal injury, inhibiting epithelial repair. Studies have found that paeoniflorin from the roots of *P. lactiflora* Pall. (Paeoniaceae), by specifically targeting the Pro169 site of PTEN protein, promotes ubiquitination and degradation and inhibits membrane localization. This activates the AKT/CREB signaling pathway independently of PI3K, leading to enhanced expression of tight junction proteins. Paeoniflorin promotes repair of ethanol-induced human gastric organoid mucosal injury and reconstructs gastric barrier function (Hu et al., 2025).

### 5.2 Activation of stem cell regeneration and repair of atrophic gastric mucosa

Atrophic gastritis (AG) is an early key stage of inflammation-to-carcinoma transition. Its core pathological features include disruption of gastric gland structure and decline in endogenous stem cell function. Activation of gastric stem cells is a key strategy for repairing atrophic gastric mucosa. Gastric organoids contain functional stem cells and can recapitulate the three-dimensional structure of gastric gland units, making them an ideal platform for studying stem cell regeneration and repair mechanisms. PEEPA-P5, a natural bioactive peptide isolated from the traditional Chinese medicinal material *P. americana* L. (Blattidae), can act as an EGF-like gastric stem cell niche molecule. It activates downstream EGFR signaling, promotes growth of normal mouse-derived gastric organoids, and upregulates Lgr5+ stem cells and various lineage differentiation markers (Li K. et al., 2024). Patient-derived gastric organoids from atrophic gastritis showed extremely low survival rates and downregulated Lgr5 expression. P5 treatment significantly improved their survival and growth rates and partially restored various cell lineage markers, achieving functional regeneration of atrophic mucosa. However, P5 showed slight inhibition of gastric organoids derived from non-atrophic gastritis patients, suggesting differential regulation of stem cells between pathological and normal states by the drug. This study was the first to demonstrate at the organoid level that a TCM metabolite can achieve functional regeneration of atrophic mucosa by activating rather than proliferating endogenous stem cells.

Notably, this study also utilized GES-1 cells to discover that P5 slowly and dynamically activates ERK and Jak-Stat1 signaling. It achieves sustained signal activation by stabilizing the EGF-EGFR complex on the cell membrane and delaying receptor endocytic degradation. Additionally, GES-1 cells were used to determine the safe concentration range of P5 (IC50 = 284.4 μmol/L), providing dose references for organoid studies. This demonstrates the value of dual-model integration.

### 5.3 Reversal of cellular homeostasis imbalance and blockade of malignant transformation

The transition of gastric mucosa from precancerous lesions to malignant transformation involves successive or simultaneous disturbances at multiple levels of homeostasis. These include imbalance of proliferation, differentiation, and apoptosis, reprogramming of energy metabolism programs, and dysregulation of redox status. Key pathways initially identified through screening in GES-1 cells require tissue-level functional validation in three-dimensional structures. This reflects the research value of gastric organoids in this field.

At the level of proliferation and differentiation regulation, aberrant activation of the STAT3 signaling pathway drives abnormal cell proliferation and chronic inflammation. In the GES-1 malignant transformation model described in Section 4.6.1, sustained STAT3 phosphorylation was preliminarily identified as a key node for TCM intervention. Building on this, using human precancerous organoids (hPCO), Hao et al. demonstrated that the natural flavonoid luteolin (Lut) from *Reseda luteola* L. (Resedaceae) can directly bind to the DNA-binding domain of the STAT3 transcription factor. It blocks its binding to the LCN2 gene promoter region, thereby significantly inhibiting the expression of the pro-tumor factor LCN2. In hPCO, lentivirus-mediated LCN2 knockdown fully recapitulated the anti-proliferative effects of luteolin, while exogenous LCN2 protein partially rescued the phenotype (Hao et al., 2025). This study confirmed the key mechanism by which luteolin reverses the precancerous state through blockade of the STAT3/LCN2 axis.

Energy metabolism reprogramming is a core feature enabling tumor cells to maintain rapid proliferation. Normal cells generate ATP mainly through mitochondrial oxidative phosphorylation, while tumor cells prefer glycolysis even under normoxic conditions, a hallmark termed the Warburg effect (Tufail et al., 2024). Despite its low ATP yield, glycolysis rapidly supplies energy and generates biosynthetic precursors for nucleotides, amino acids and lipids (Jaworska et al., 2023). Therefore, forcing restoration of oxidative phosphorylation does not merely supplement energy but forces tumor cells to switch from a proliferation-supporting glycolysis-dependent mode to an ATP-producing oxidative phosphorylation-dependent energy mode. This deprives them of the material basis required for rapid proliferation (Bao et al., 2026). The study on Lianqiao Wendan Decoction (LQWDD; composed of *Forsythia suspensa* (Thunb.) Vahl [Oleaceae], *P. ternata* (Thunb.) Makino [Araceae], *B. tuldoides* Munro [Poaceae], *C. aurantium* L. [Rutaceae], *C. reticulata* Blanco [Rutaceae], *W. cocos* (F.A.Wolf) Ryvarden and Gilb. [Polyporaceae], and *G. uralensis* Fisch. ex DC. [Fabaceae]) provides evidence supporting this strategy. In human gastric high-grade intraepithelial neoplasia (HGIN) tissues, mitochondria exhibited swelling and cristae rupture, and the integrity of the oxidative phosphorylation pathway was significantly inhibited. Lianqiao Wendan Decoction repaired oxidative phosphorylation function by upregulating the mitochondrial complex I-related protein ALDH3A1 and stabilizing the protein NDUFS4. Its potential active metabolite quercetin activated the AMPK signaling pathway, phosphorylating downstream PGC-1α to promote mitochondrial biogenesis and oxidative metabolism-related gene expression. This further enhanced mitochondrial oxidative respiration and inhibited the growth of patient-derived gastric cancer organoids (Chi et al., 2025). This study comprehensively employed proteomics, metabolomics, and organoid models to systematically reveal the mechanism by which a TCM formula inhibits tumor cell proliferation at the metabolic level through reversal of metabolic reprogramming.

Redox homeostasis is an important dimension of cellular homeostasis. Ferroptosis is an iron-dependent form of programmed cell death whose core mechanism involves excessive accumulation of lipid peroxides leading to cellular oxidative damage. In recent years, ferroptosis has been confirmed to be closely associated with the development of gastric polyps. Studies have found that Weifuchun (WFC) dose-dependently inhibited the growth of gastric fundic gland polyp organoids and regulated glandular structural disorganization. It modulated the expression of the key ferroptosis inhibitory protein glutathione peroxidase 4 (GPX4) and acyl-CoA synthetase long-chain family member 4 (ACSL4), leading to accumulation of lipid peroxides and triggering ferroptosis in polyp cells. This effect was reversed by the ferroptosis-specific inhibitor Ferrostatin-1 (Yue et al., 2025). This discovery provides a mechanistic basis for TCM-induced ferroptosis to intervene in the benign hyperplasia stage of the inflammation-to-carcinoma transition process. Notably, ferroptosis is not an isolated cell death event. As described in Sections 4.1, 4.2, the Nrf2 pathway is central to maintaining cellular redox homeostasis, whereas NLRP3/Caspase-1/GSDMD is a key execution axis of inflammatory cell death. Recent studies indicate that these two pathways also interact closely in the context of ferroptosis: the Nrf2/GPX4 antioxidant axis limits lipid peroxidation and ROS-mediated NLRP3 inflammasome activation; GPX4 or glutathione depletion promotes lipid peroxide accumulation, thereby amplifying NLRP3/Caspase-1/GSDMD pyroptotic signaling; conversely, NLRP3 activation and IL-1β release further increase oxidative stress, sensitizing cells to ferroptosis (Yan et al., 2025). This suggests that WFC-induced ferroptosis in gastric polyp organoids may not be mediated solely by the GPX4/ACSL4 axis, but may potentially interact with Nrf2-dependent redox control and NLRP3-related inflammatory cell death. Future validation of these crosstalk nodes in organoid models will help to more comprehensively understand the mechanistic features of multi-pathway TCM interventions in inflammation-to-carcinoma transition.

Collectively, these three lines of evidence demonstrate that gastric organoids can validate, in three-dimensional tissue structures, the molecular targets suggested by GES-1 cell models—from STAT3/LCN2-driven abnormal proliferation, to AMPK/OXPHOS-mediated metabolic reprogramming, to GPX4/ACSL4-regulated redox imbalance. They also confirm that intervention at these targets indeed reverses pathological phenotypes at the tissue level.

### 5.4 Evaluation of antitumor effects

Gastric cancer organoids retain the genetic characteristics and heterogeneity of patient tumors. They faithfully reflect molecular subtypes, mutational profiles, and drug response features. Compared with traditional two-dimensional cell lines and xenograft models, they combine high-throughput feasibility with clinical relevance. They serve as an ideal bridge between basic research and translational application. In the study by Hao et al., patient-derived gastric cancer organoid models were used to validate the anti-tumor effects of luteolin. The results showed that luteolin dose-dependently inhibited the growth of gastric cancer organoids. Gastric cancer organoids were more sensitive to 20 μM luteolin than normal gastric tissue organoids, providing experimental support closer to the actual tumor environment for its clinical translation (Hao et al., 2023). This study suggests that luteolin may have differential efficacy among different molecular subtypes of gastric cancer, laying a foundation for evaluating TCM anti-tumor effects and individualized therapy.

Furthermore, combined with the mechanistic study in Section 5.3 demonstrating that this metabolite blocks malignant transformation of precancerous lesions in hPCO models through the STAT3/LCN2 axis (Hao et al., 2025), these findings together indicate that luteolin possesses intervention potential at different stages of inflammation-to-cancer transition. It can both inhibit the initiation of malignant transformation at early stages and suppress the growth of already formed tumors at advanced stages.

### 5.5 Application prospects of gastric organoid models in TCM research

The gastric organoid model types mentioned above have been well established at the technological level. However, their systematic application in the TCM field is still in its infancy. Current TCM studies have largely focused on patient-derived gastric precancerous lesion organoids and gastric cancer organoids. Reports on *H. pylori*-infected organoids and chemically induced organoids are extremely limited. This section provides an overview of the above gastric organoid models, with emphasis on their application potential in TCM research.

*H. pylori*-infected gastric organoids are established by microinjecting *H. pylori* into the luminal surface of organoids or by co-culture. These models can simulate *H. pylori* colonization, persistent infection, and host cellular responses in the gastric mucosal epithelium *in vitro* (Hofer et al., 2025; Yu B. M. et al., 2025). They are useful for observing the infection effects of *H. pylori* on different epithelial cell types. They are also suitable for screening TCM active metabolites that inhibit *H. pylori* colonization or attenuate epithelial damage induced by the bacterium.

In existing studies, deoxycholic acid or MNNG has been added to culture media to induce mouse gastric organoids. This approach simulates lesions such as intestinal metaplasia under controlled conditions (Jin et al., 2022; Li Y. et al., 2024). Although these models have been established predominantly in murine organoids, their methodological feasibility has been demonstrated. They also circumvent the inherent inter-individual heterogeneity of patient-derived organoids. These features provide a controllable *in vitro* platform for observing the intervention effects of TCM on mucosal injury and metaplasia induced by specific pathogenic factors. In TCM research, such models can be used to evaluate the protective effects of TCM active metabolites against bile acid- or chemical carcinogen-induced gastric mucosal injury, as well as their potential to reverse gastric precancerous lesions. Chemically induced organoids are not restricted to the intestinal metaplasia stage; after prolonged exposure to inducing agents, they can also develop dysplasia-like phenotypes. When patient-derived samples are unavailable, they can serve as a controllable alternative.

Patient-derived gastric organoids are currently the most widely used type in TCM research. Gastric precancerous lesion organoids retain the histopathological features of donor tissues, including atrophy, intestinal metaplasia, or dysplasia. They allow verification of the efficacy of TCM in reversing precancerous lesions at the tissue level. Gastric cancer organoids, derived from surgically resected gastric cancer tissues, preserve the genetic heterogeneity and drug response characteristics of patient tumors (Yan et al., 2018). Compared with traditional two-dimensional cell lines, gastric cancer organoids more faithfully reflect the molecular subtypes, mutational profiles, and drug sensitivity of tumors. They have been widely used in the pharmacodynamic evaluation of chemotherapeutic agents and novel anti-tumor metabolites (Zhao Y. et al., 2024). In the TCM field, gastric cancer organoids hold promise for individualized assessment of the anti-tumor effects of TCM formulas or active metabolites. They can provide experimental evidence for precision therapy in TCM (Kan et al., 2025).

These three organoid types show clear functional division in TCM research. Infection- and induction-based organoids offer controllable mechanisms for target screening, whereas patient-derived organoids enable individualized pharmacodynamic validation. They address complementary questions—mechanism of action versus individual drug response. With further standardization and broader TCM involvement, these models should play a greater role in TCM research on gastric mucosal inflammation-to-carcinoma transition.

## 6 Discussion

### 6.1 Evidence level and methodological quality assessment

To enable tiered interpretation of evidence strength across the included studies, we established a two-dimensional evaluation framework based on the preclinical evidence pyramid and *in vitro* reporting guidelines (QUIN, SciRAP). The evidence level reflects the clinical relevance and tissue fidelity, with three hierarchical levels—A, B, and C. Tier A, the highest level, includes patient-derived organoids or primary human samples validating drug effects in three-dimensional tissue architectures, conferring the greatest translational value. Tier B includes genetically engineered or chemically induced organoids allowing controlled observation of tissue-level pathological alterations driven by specific pathogenic factors. Tier C, the foundational level, covers monolayer cultures of immortalized cell lines such as GES-1, suitable for molecular target screening and signaling pathway dissection but lacking tissue microenvironmental context; conclusions require validation in higher-tier models.

To ensure grading consistency, the evidence level was determined by the highest-level *in vitro* model used. Studies using only GES-1 cells were classified as Tier C, regardless of pharmacological inhibitors or animal experiments. When multiple *in vitro* models were used, the highest level was assigned. This framework assesses *in vitro* model fidelity, not overall evidence chain completeness; thus, animal experiments alone do not elevate the *in vitro* evidence level. Unlike QUIN and SciRAP, which assess bias and reporting quality, this framework focuses on model selection and similarity to human gastric tissue; the two are complementary. Methodological quality was assessed independently, focusing on drug intervention type and concentration range, control setup, intervention duration, and target validation methods (Table 1).

Overall, the included studies exhibit a progressive trend toward increasing model complexity, yet several common limitations remain evident. Tier C studies constitute the majority of this review. These studies generally provide complete dose–response data and appropriate positive controls, demonstrating high reliability for preliminary target screening; however, their pathway validation relies predominantly on pharmacological inhibitors, lacking genetic manipulations such as CRISPR/Cas9-mediated gene knockout or siRNA knockdown, which constrains the strength of causal inference. Notably, no Tier B studies highly relevant to the topic of this review were included. Tier A studies exhibit the highest methodological rigor, featuring patient-derived organoids, direct target-binding evidence, and head-to-head comparisons with clinical reference drugs; nevertheless, common constraints include small sample sizes, lack of stromal/immune microenvironmental components, and insufficient pharmacokinetic data. These observations indicate that, although the field is gradually evolving toward more physiologically relevant models, the overall evidence base remains predominantly exploratory. Future efforts should strengthen genetic validation, active ingredient identification, and multicenter cohort studies to enhance the translational validity of traditional Chinese medicine interventions for gastric precancerous lesions.

Furthermore, although this review searched PubMed, Web of Science, and SinoMed, only English-language publications were retained. High-quality studies in Chinese core journals may therefore have been omitted, resulting in literature selection bias. As this article is a narrative review focusing on model selection logic rather than exhaustive evidence synthesis, the search was not extended to Chinese databases. Conclusions should be interpreted cautiously in light of this selection bias, and future reviews should include Chinese databases to obtain more comprehensive evidence.

### 6.2 Current applications of *in vitro* models in mechanistic research on inflammation-to-carcinoma transition

This review compares the applications of GES-1 monolayer cells and gastric organoids in TCM intervention research on gastric mucosal inflammation-to-carcinoma transition. The core finding is that the two model types play different but complementary roles in the evidence chain (Table 2). GES-1 cells are suitable for initial screening of molecular targets and safety evaluation of active metabolites. Gastric organoids are suitable for tissue-level functional validation. Unlike previous reviews that separately focused on organoid culture techniques, specific signaling pathways, or the pharmacodynamic mechanisms of single TCM formulas, this review, from the perspective of model selection and integration, proposes a hierarchical research framework spanning different stages of the Correa cascade.

**TABLE 2:** Comparison of GES-1 cell monolayer and gastric organoid models for TCM research on the gastric inflammation-to-carcinoma transition.

Feature	GES-1 cell	Gastric organoid
Structural dimension	Two-dimensional (2D)	Three-dimensional (3D)
Cellular composition	Single immortalized gastric epithelial cell line	Multiple cell types (stem cells, chief cells, parietal cells, etc.)
Genetic background	Uniform	Retains donor genetic features
Disease stage modeled	Acute injury, early-stage inflammation	Chronic inflammation, atrophy, intestinal metaplasia, dysplasia, gastric cancer
Typical injurious inducers	Ethanol, H_2_O_2_, LPS, NSAIDs	*H. pylori*, MNNG, bile acids, mycotoxins
Culture duration	Short	Long
Primary applications	Acute injury mechanism screening, safety evaluation of active metabolites, high-throughput drug screening	Tissue-level validation, personalized efficacy evaluation, chronic progression modeling, gene editing
Advantages	Easy handling, high reproducibility, cost-effective	High physiological relevance, retains tissue architecture, models stem cell behavior
Limitations	Lack of 3D structure, simple cell-cell interactions, no stem cell niche	Lack of immune/neural/vascular components, high cost, low throughput
Complementary role in TCM research	Identify active metabolites and primary targets	Validate tissue-level effects, assess clinical translational potential

In current research, the construction strategies for GES-1 cell models are becoming increasingly sophisticated. Early studies predominantly used ethanol, hydrogen peroxide, lipopolysaccharide, and similar agents to stimulate GES-1 cells to establish inflammation models for screening anti-inflammatory active metabolites of TCM. This classical model is operationally simple and high-throughput, playing a key role in preliminarily identifying the active material basis of TCM formulas. However, gastric mucosal inflammation-to-carcinoma transition is a multi-step process involving *H. pylori* infection, microenvironmental changes, and epigenetic regulation. Single-stimulus models cannot fully reflect its complexity. Some studies have begun using MNNG to simulate environmental carcinogen exposure and establish gastric precancerous lesion cell models (Liu S. et al., 2025). Others have used deoxycholic acid (DCA) to induce GES-1 cell differentiation toward intestinal metaplasia (Jin et al., 2022; Wang et al., 2021). These approaches simulate early molecular events in the transition of normal gastric mucosal cells toward cancer cells. Although such models have obvious limitations in reflecting tissue-level pathological progression, they still have certain reference value for initial screening of candidate molecules affecting early transformation events. Furthermore, some studies have placed GES-1 cells in co-culture with immune cells or in conditioned media environments. They found that inhibition of M2 macrophage polarization attenuates the malignant phenotype of GES-1 cells, revealing that BRD4 influences epithelial cell fate through regulation of the immune microenvironment (Zhu et al., 2024). Additionally, extracellular vesicles derived from *H. pylori*-infected GES-1 cells can induce malignant transformation in uninfected cells. This finding suggests that inflammatory damage can spread through bystander effects and provides a new perspective for understanding the propagation mechanisms of inflammation-to-carcinoma transition (González et al., 2022). These studies indicate that the application of GES-1 cell models is expanding from single acute injury protection mechanism analysis to multiple research scenarios including chronic stimulation responses and microenvironmental interactions. However, their core advantage remains in molecular mechanism screening under acute injury conditions.

Compared with immortalized cell lines such as GES-1, gastric organoid technology provides a platform closer to the physiological state for inflammation-to-carcinoma transition research. They can three-dimensionally recapitulate glandular structures, cell lineage differentiation, and microenvironmental interactions. And they are the preferred model for simulating chronic accumulation and malignant transformation at the tissue level. A recent important advance utilized patient-derived atrophic gastritis organoids and sequentially knocked out key genes including RUNX3, SMAD4, and TP53 using CRISPR/Cas9 technology. This successfully modeled the transformation process from atrophic gastritis to intestinal metaplasia and even tumors *in vitro*. This study not only confirmed that Wnt signaling is critical for maintaining atrophic mucosal homeostasis but also revealed BMP/SMAD4 signaling-dependent intestinal differentiation pathways. It also showed that concurrent deletion of TP53 and SMAD4 confers tumorigenic capacity by relieving p21-induced senescence (Yoshihiro et al., 2025). This study precisely recapitulated key molecular events in the Correa cascade, providing a mechanistic research platform for validating TCM interventions. Review articles have also noted that future integration of gastric organoids with single-cell omics and real-time imaging technologies will enable more refined analysis of the roles of immunosuppression and metabolic reprogramming in inflammation-to-carcinoma transition (Liu C. et al., 2025). It should be noted that research on gastric organoids in the TCM field is still in its infancy. Existing literature is largely concentrated on human gastric precancerous lesion organoids and gastric cancer organoid models. Reports on *H. pylori*-infected organoids, bile acid-or carcinogen-induced organoid models in TCM intervention research are extremely limited. This reflects the current developmental stage of the field rather than selective omission by this review. These model types are technologically mature at the organoid level, but their application in TCM research awaits systematic development.

Based on the existing literature, GES-1 cell models and gastric organoid models exhibit clear complementarity in TCM prevention and treatment research on gastric mucosal inflammation-to-carcinoma transition. Two-dimensional cell models are characterized by low cost, short cycle time, and suitability for high-throughput screening. They are ideal tools for active metabolite identification and potential target prediction, and can also be used for toxicity evaluation of TCM active metabolites on gastric mucosal epithelial cells under normal physiological conditions, providing a safe concentration range reference for subsequent organoid and animal experiment dosing design. Gastric organoid models allow observation of tissue-level drug effects in a three-dimensional environment closer to *in vivo* conditions. They can employ gene editing technologies to determine the necessity of specific molecular targets, making them more suitable for functional validation and mechanistic deepening of screening results. Based on the respective advantages and division of labor of the two models, we have summarized a corresponding model selection framework (Table 3). This framework follows a stepwise progression: from GES-1-based molecular screening, to organoid-based tissue validation, and then to individualized evaluation using patient-derived models. This logic is guided by the pathological features of each stage of the Correa cascade and the requirements for *in vitro* modeling.

**TABLE 3:** Model selection framework for *in vitro* studies across Correa cascade stages.

Disease stage	Recommended model	Common inducers	Major readouts	Suitable research purpose
Acute mucosal injury	GES-1 cells	Ethanol, H_2_O_2_, LPS, NSAIDs	Cell viability, ROS, MDA, SOD, GSH, inflammatory cytokines, apoptosis rate	Initial screening of active metabolites, acute mechanism elucidation
Chronic atrophic gastritis	GES-1 cells/Gastric organoids	GES-1 cells: MNNG; Long-term *H. pylori* infection Gastric organoids: MNNG; Long-term *H. pylori* infection; Patient-derived atrophic gastritis tissues	GES-1 cells: Cell viability, apoptosis rate, inflammatory markers, CDX2, SOX2, MUC5AC Gastric organoids: Glandular structure, MUC5AC/MUC6, proliferation marker, stem cell function	Molecular screening and tissue-level validation of atrophy reversal mechanisms
Intestinal metaplasia	GES-1 cells/Gastric organoids	Deoxycholic acid	GES-1 cells: Cell viability, CDX2, MUC2, KLF4, VIL1, SOX2, MUC5AC Gastric organoids: Glandular structure, CDX2, MUC2, villus architecture	Molecular screening and tissue-level validation of intestinal metaplasia reversal
Dysplasia	Gastric organoids	Directly derived from patient tissues or chemically induced organoids (e.g., long-term MNNG or DCA exposure)	Glandular atypia, proliferation marker, p53	Efficacy validation of TCM in reversing precancerous lesions
Gastric cancer	Patient-derived gastric cancer organoids	Directly derived from patient tumor tissues	Organoid size, viability, drug sensitivity, proliferation marker	Anti-tumor efficacy evaluation, personalized therapy prediction

### 6.3 Limitations and future directions

These *in vitro* studies largely rely on pharmacological inhibitor to infer pathway necessity and lack orthogonal validation such as gene knockout or knockdown, potentially affecting conclusion robustness. Furthermore, both *in vitro* models have multiple limitations (Zheng et al., 2026). The immortalized nature of GES-1 cells may cause non-physiological metabolic and signaling alterations. Monolayer culture loses the three-dimensional architecture of native gastric mucosa and cannot recapitulate cell–cell or cell–extracellular matrix interactions. It also cannot reflect the differentiation lineages of multiple cell types coexisting in gastric gland units. Although gastric organoids better resemble *in vivo* tissue structure, they mainly contain epithelial components and lack key microenvironmental elements such as immune cells, blood vessels, stromal fibroblasts, and nerves, hindering simulation of epithelial–microenvironment interactions during inflammation-to-carcinoma transition. Additionally, high cost, long cycle time, and low throughput limit their scalability for large-scale drug screening.

Given these limitations, both models should be positioned as tools rather than endpoints. Their value lies in generating testable mechanistic hypotheses, not predicting clinical efficacy. Clinical translation still requires animal and human validation. Therefore, GES-1 cells should be extended to co-culture systems with immune and stromal cells, shifting from isolated target cell studies to multicellular interactions. Gastric organoids should integrate immune, stromal, and vascular components for dynamic microenvironment reconstruction, moving from structural to functional simulation. Specifically, future research should shift from purely epithelial organoids to multicomponent dynamic systems. For example, organoid-immune cell co-culture can incorporate macrophages, dendritic cells, or T cells to reconstruct epithelial-immune crosstalk during *H. pylori* infection and chronic inflammation (Wang J. et al., 2025); air-liquid interface culture can better preserve mucus secretion, epithelial polarization, and host-microbe interactions; microfluidic organ-on-a-chip can integrate perfusion, mechanical forces, oxygen gradients, and spatial separation of epithelial, stromal, vascular, and immune components (Hofer et al., 2025). Combining gastric organoids with organ-on-a-chip and immune co-culture systems may reconstruct key features of the inflammation-to-carcinoma microenvironment, including chronic cytokine exposure, immune evasion, angiogenesis, and stromal remodeling, thereby providing a more predictive platform for evaluating TCM interventions before animal and clinical studies. *In vitro* mechanistic findings require multilevel validation via animal models, organoids, and clinical samples to bridge basic and clinical research.

## Glossary

TCM: Traditional Chinese Medicine
GES-1: Human gastric mucosal epithelial cell line
MNNG: N-methyl-N′-nitro-N-nitrosoguanidine
DCA: Deoxycholic acid
ROS: Reactive oxygen species
SOD: Superoxide dismutase
GSH: Glutathione
MDA: Malondialdehyde
HO-1: Heme oxygenase-1
NQO1: NAD(P)H quinone oxidoreductase 1
NF-κB: Nuclear factor-kappa B
LPS: Lipopolysaccharide
TLR4: Toll-like receptor 4
NLRP3: NOD-like receptor family pyrin domain containing 3
ASC: Apoptosis-associated speck-like protein containing a CARD
IL-6: Interleukin-6
IL-1β: Interleukin-1 beta
TNF-α: Tumor necrosis factor-alpha
PI3K: Phosphoinositide 3-kinase
AKT: Protein kinase B
MAPK: Mitogen-activated protein kinase
ERK: Extracellular signal-regulated kinase
JNK: c-Jun N-terminal kinase
EGFR: Epidermal growth factor receptor
EMT: Epithelial–mesenchymal transition
STAT3: Signal transducer and activator of transcription 3
JAK2: Janus kinase 2
CDX2: Caudal-type homeobox transcription factor 2
GPL: Gastric precancerous lesion
hPCO: Human precancerous organoids
GPX4: Glutathione peroxidase 4
ACSL4: Acyl-CoA synthetase long-chain family member 4
